# Computational Study of Complex Formation between Hyaluronan Polymers and Polyarginine Peptides at Various Ratios

**DOI:** 10.1021/acs.langmuir.3c01318

**Published:** 2023-09-29

**Authors:** Natalia Kulik, Babak Minofar, Adam Jugl, Miloslav Pekař

**Affiliations:** † Laboratory of Photosynthesis, Institute of Microbiology, Czech Academy of Sciences, Novohradská 237 - Opatovický mlýn, 379 01 Třebon, Czech Republic; ‡ Faculty of Science, University of South Bohemia, Branišovská 1760, 370 05 České Budějovice, Czech Republic; § Faculty of Chemistry, Brno University of Technology, Purkyňova 118, 612 00 Brno, Czech Republic

## Abstract

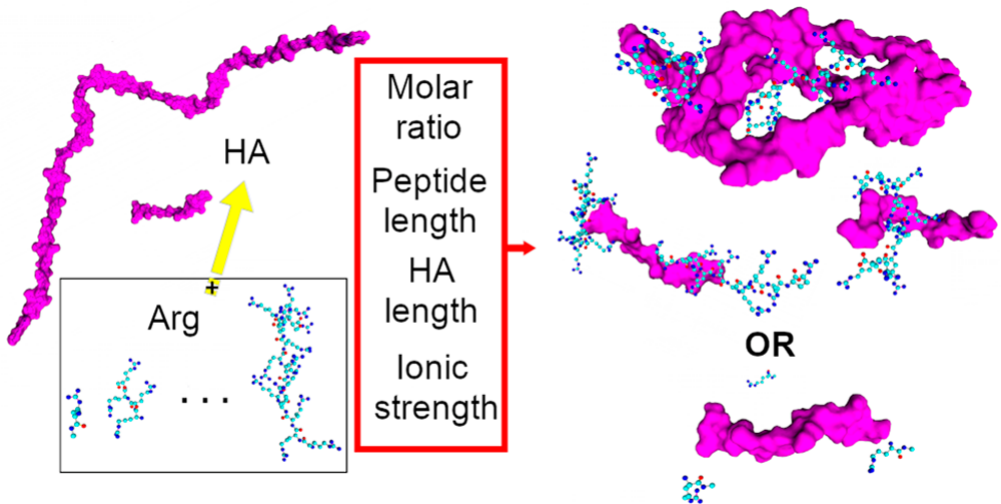

Hyaluronic acid, a naturally occurring carbohydrate biopolymer in human tissues, finds wide application in cosmetics, medicine, and material science. Its anionic properties play a crucial role in its interaction with positively charged macromolecules and ions. Among these macromolecules, positively charged arginine molecules or polyarginine peptides demonstrate potential in drug delivery when complexed with hyaluronan. This study aimed to compare and elucidate the results of both experimental and computational investigations on the interactions between hyaluronic acid polymers and polyarginine peptides. Experimental findings revealed that by varying the length of polyarginine peptides and the molar ratio, it is possible to modulate the size, solubility, and stability of hyaluronan–arginine particles. To further explore these interactions, molecular dynamics simulations were conducted to model the complexes formed between hyaluronic acid polymers and arginine peptides. The simulations are considered in different molar ratios and lengths of polyarginine peptides. By analysis of the data, we successfully determined the shape and size of the resulting complexes. Additionally, we identified the primary driving forces behind complex formation and explained the observed variations in peptide interactions with hyaluronan.

## Introduction

Hyaluronic acid (HA) is a polymer formed from the basic disaccharide units d-glucuronic acid (GlcA) and *N*-acetyl-d-glucosamine (GlcNAc). It is a biopolymer with a wide range of naturally occurring molecular masses from several hundreds to 1 × 10^7^ g/mol with versatile functions.^1,2^

The interactions between HA and amino acids, particularly arginine (Arg) residues, play a significant role in the biological activities. One of the well-known functions of HA is its interaction as a ligand with the CD44 receptor where arginine residues are involved.^3^ HA also participates in cell proliferation, motility, and invasion where the interaction of HA with CD44 receptor and RHAMM protein is involved.^4^

The interaction between the negatively charged carboxylic groups of HA and positively charged residues, with a preference for Arg residues, is crucial for the functioning of hyaluronan synthase.^5^ Moreover, nanoparticles incorporating Arg peptides and HA demonstrate great potential as complex structures for drug delivery purposes.^6−8^

The ability of HA to interact with the CD44 surface and with positively charged peptides was found to determine the potential of their use as biodegradable decoration of the surface of drug carriers.^9^ This is particularly significant considering the advantageous properties of Arg peptides consisting of 8–9 residues, which are known for their efficient cellular penetration abilities.^10^

The polyelectrolyte nature of HA, coupled with its ability to form hydrogels and the presence of carboxyl and hydroxyl groups that can be easily modified, makes it a versatile biomaterial.^2,11^ These properties enable its application not only in drug delivery systems but also in diverse fields such as cosmetics, tissue engineering, and organ implantation.^2,12^

There is a scarcity of studies focusing on the interactions between HA and amino acids as well as their computer modeling. However, a research endeavor utilizing the density functional theory method has investigated the interactions between HA and arginine and lysine.^13^ The authors observed a decrease in viscosity as the concentration of arginine (Arg) increased. Based on this observation, they speculated that the Arg residues have the ability to bind HA molecules together, causing a change in their conformation toward a more bent structure. It is important to note, however, that the study did not investigate long interaction times between HA and amino acid residues, and the modeled systems involved only small HA–Arg complexes consisting of 1–2 HA units.

To comprehensively investigate the interactions between HA and Arg, an experimental study was conducted. This study was performed by analyzing the interactions between HA and polyarginine peptides of varying lengths and molar ratios by ultrasonic spectroscopy and isothermal titration calorimetry.^14^ Molar ratio is the number of HA units divided by the number of Arg residues as introduced in ref (14). The findings unveiled that the interactions between hyaluronan and arginine oligomers manifested specifically in oligomers with 8 monomer units or longer chains. However, it was observed that these interactions were effectively suppressed in the presence of a sufficiently high ionic strength, which contrasted with their occurrence in water. Based on the analysis, it was proposed that the interplay of hydration forces and electrostatic interactions, along with electrostriction, hydrogen bonding, and hydrophobic contacts between desolvated parts of (macro)molecules, collectively contributed to the observed interactions. Notably, the specific conformations adopted by hyaluronan and arginine oligomers were also found to play a significant role in the interactions.^14^

In order to gain insights into the dynamics of HA–polyarginine complex formation and to assess the interactions between HA and polyarginine peptides of varying lengths, this study employed computational modeling and molecular dynamics simulations (MD). Furthermore, the findings of this theoretical investigation were validated by comparing them with the experimental results.^14^

## Materials and Methods

### Modeling of Polyarginine Peptides, HA, and HA–Arg Complexes

Polyarginine peptides of different lengths were built with YASARA^15^ and minimized in a vacuum by the standard protocol.^16^ For MD simulations of peptides without HA we used extended peptide structures as initial; only for 14 Arg polypeptides we use extended and helical structures as initial. HA molecules were built with Glycam builder (http://glycam.org). The force-field parameters of the GLYCAM 06 force field downloaded from Glycam builder^17^ were converted to GROMACS topology and coordinates by the amb2gmx.pl script.^18^

### Composition of the System

HA–polyarginine peptide systems were built with the Packmol package.^19^ HA and peptides were randomly placed in the simulation cells. The modeled initial configuration from Packmol was accepted if the following requirements were fulfilled: components of the system (HA, peptides) are far enough from each other and do not form hydrophobic, stacking, or HB interactions; peptides are distributed through the entire box. The required numbers of particles for particular systems and concentrations were calculated according to the published data.^14^ The weight concentration of HA in the aforementioned work was 0.1%. For MD simulations, we used 0.1% w/w (systems with 1HA25) and increased this to 0.2% w/w (systems with 2HA25). The 1HA25 molecule consists of 25 hyaluronic acid units with a molecular mass of 9 kDa.

Conducting molecular dynamics simulations at the atomic level for high molecular weight HA can be computationally demanding and time-consuming. To capture the essential characteristics of complex formation between higher molecular weight HA and polyarginine peptides, we employed MD simulations with two HA molecules, each consisting of 25 units (18 kDa). To maintain a realistic environment resembling experimental conditions, all the simulations were performed with approximately 0.01 mM NaCl (with the ions utilized solely for system neutralization and pH adjustment to 7), which corresponded to the conditions found in the experiments.^14^ A particular system, denoted as 2HA25-5Arg10, was simulated under a higher salt concentration of 100 mM NaCl.

### MD Simulation

MD simulations were run using the GROMACS 5.1.2 package.^20^ GLYCAM 06 force field parameters^17^ were used for HA, and AMBER 03 force field parameters^21^ were used for polyarginine peptides, water, and ions. Water molecules (the TIP3P water model)^22^ and neutralizing ions were added by the GROMACS program. A cubic box was used to set the periodic boundary conditions. The minimum size of the box was 19 nm in each direction. Polyarginine peptides were modeled as poly-l-arginine hydrochloride; hence, a corresponding number of Cl ions were added to each system to neutralize the positively charged Arg residues. GlcA in HA was modeled in deprotonated form, and a corresponding number of Na ions were added by GROMACS to each system to neutralize GlcA. Simulations were run as an isothermal–isobaric ensemble: a constant number of particles; a constant pressure of 1 bar, controlled by a Parrinello–Rahman barostat;^23^ and a constant temperature of 300 K, corrected by a velocity-rescaling temperature coupling thermostat.^24^ The particle mesh Ewald algorithm was used to calculate long-range electrostatics.^25^ MD simulations were conducted for a duration of 50–200 ns. The length of the simulations was determined based on the time necessary for system equilibration, which was established by observing a stable root-mean-square deviation (RMSD) and the formation of a consistent and stable complex in the case of HA–polyarginine systems (minimal possible number of clusters stable for 20 ns). A list of analyzed systems is included in the Supporting Information (Table S1). Some simulations were repeated several times, either due to the interaction with the image of itself under the periodic boundary conditions (2HA25-5Arg10) or in order to validate the results (2HA25-3Arg14). Data for representative MD simulation for systems with multiruns are reported if not stated otherwise. The pyranose ring oxygen atoms of carbohydrates were chosen as reference atoms for the calculation of the radial distribution function (RDF) of HA. Reported average parameters are calculated for equilibrated MD simulation times. Hydrophobic interactions are calculated by YASARA^15^ with distance approach, assuming that distance between atoms of hydrophobic groups should be below 0.7 and 0.4 nm between closest hydrogens atoms.

### Calculation of Free Energy of Binding

To estimate the free energy of binding, the MM-PBSA method in GROMACS was used.^26^ This method calculates binding free energy according to eq 1:
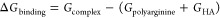
1In this equation, *G*_complex_, *G*_polyarginine_, and *G*_HA_ represent the energies of the complex, polyarginine peptide, and HA in solution (water), respectively. To calculate these free energies, a combination of averaged molecular-mechanics energies in a vacuum (including van der Waals and electrostatic contributions) and averaged free energies of solvation (including polar and apolar components) are considered. The solvation energy consists of both polar and apolar contributions. The polar contribution is defined on the basis of the electrostatic potential of the solvent and solute. There are several models for the calculation of apolar energy. We selected the SASA-only (SASA: solvent accessible surface) and SASA-WCA models (WCA: Weeks–Chandler–Andersen) for the calculation. According to the statistics, the SASA apolar solvation model, which takes into account cavity formation, showed a good correlation with the experimental data. However, our system was highly charged and hydrophilic—hence, attractive van der Waals interactions (hydrophobic input) between the solute and solvent were added to the calculations (WCA solvation model).

Peptides with various orientations to HA polymers were chosen to calculate the binding free energy. Snapshots corresponding to these orientations were selected from the complete molecular dynamics simulation. However, the portion of the MD simulation analyzed for this purpose was limited to 10 ns. It should be noted that certain potential binding poses might not have been represented accurately due to undersampling during the MD simulation. In other words, the specific orientation required for binding may not have been preserved throughout the entire 10 ns duration of the simulation. The method employed in this study to calculate the free energy is based on a one-simulation approach that does not account for the internal potential energy of the molecule or the entropic cost. The default parameters for energy calculation were used as described by Kumari et al.^26^ Only the value for the solute dielectric constant was changed to 20 (as recommended for highly charged solutes^27,28^), and the nonlinear method for polar solvent energy calculation was used. Different ionic strengths of the system 2HA25-5Arg10 (MD simulation run2) were modeled by changing the dielectric constants of the solvent (20, 30, 40, 50, and 60).

## Results and Discussion

### Stability of HA–Polyarginine Complexes during MD Simulations

**Figure 1:**
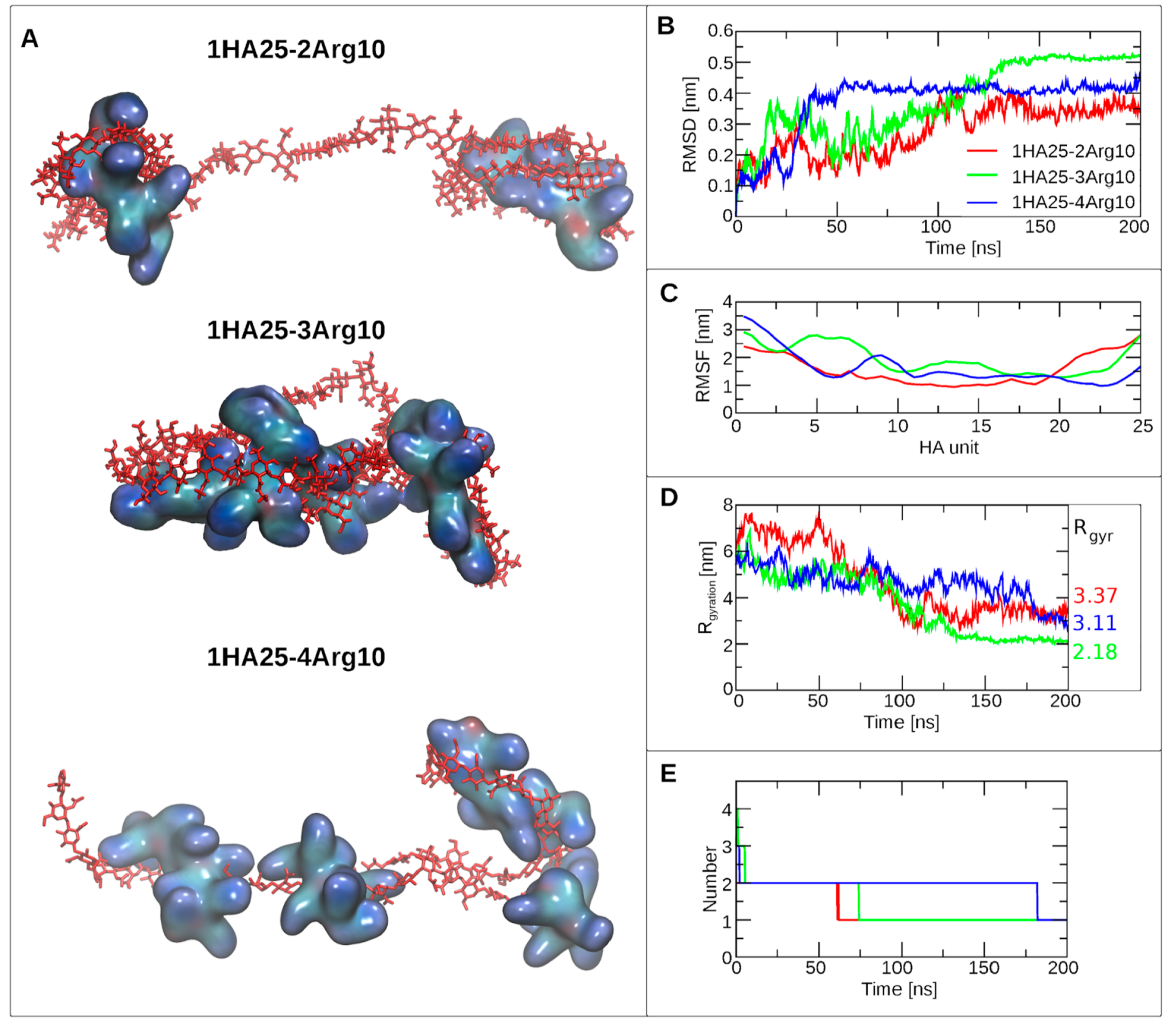
Analysis focused on the complexes formed by 1HA25 and 10 amino acid residue long polyarginine peptides. (A) Shape of the particles formed at the end of MD. HA is colored in red, while polyarginine peptides are depicted as a surface representation and colored based on the atom name The complex is labeled above in the picture. (B) RMSD of the HA molecule during MD simulations. Legend is the same for (B–E). (C) RMSF of HA molecule during MD. (D) Radius of gyration (*R*_gyration_) of HA–polyarginine peptide complexes. The average values for equilibrated complexes (at the 180–200 ns time interval) are depicted on the right side, represented by the color of the line. (E) Number of clusters formed during MD by HA and polyarginine peptides.

The stability of the systems was analyzed by calculating the RMSD of HA (Figures S1–S3, Table S1, and Figure 1A,B), the sizes of the formed clusters (Figures S4 and 1E), and root-mean-square fluctuation (RMSF). RMSD and cluster size were used to determine the equilibration time for each system (Table S1). The system was equilibrated if RMSD was stable in time (no large fluctuations), and formation of HA–polyarginine clusters with a minimal number of possible dissolved peptides was observed for a time period of at least 20 ns. For some systems (1HA4-10Arg1) formation of a stable number of clusters was never observed, and for other systems (2HA4-6Arg10, 2HA25-3Arg14 run2) the stable number of clusters was higher than 1 (Figure S4).

Dynamics of complex formation depends on the number of amino acid residues in the peptides and the molar ratio. Monoarginine residues did not form stable complexes with HA, as the number of clusters during MD fluctuates from 7 to 11 (see Figure S4H). The experiments made in ref (14) indicated that no interactions were observed between HA molecules of different molecular weights and polyarginine peptides with a length of up to 4 residues. Modeling results showed that monoarginine residues were able to form HBs with HA (Table S2) but confirmed that the lifetimes of the HA–Arg complexes were too short.

The time required to form a stable peptide–HA complex depends on the length of HA, the length of the peptides, and the concentration of HA and polyarginine. At a concentration of 0.1% w/w, HA25 formed a stable complex with peptides after 180 ns of MD simulation (Figure 1E). With the increase of HA concentration to 0.2%, equivalent to 2HA25, a shorter time required for a stable HA–polyarginine complex formation is 100 ns (Figure S4). This occurred because the higher concentration increased the probability of the molecules coming into close proximity and interacting with each other.

The formation of complexes in the systems took a longer time when a higher number of peptides were present while maintaining the same molar ratio. The formation of a stable HA–peptide complex for molar ratios of 1–1.04 was slowest in the solution with shorter peptides (2HA25-12Arg4, Figure S4).

Differences in the dynamics of HA–polyarginine peptide complex formation were observed between small peptides (Arg4) and long peptides (Arg14). When it came to polyarginine peptides with 4 residues, their adsorption at the HA surface was slower, and they could be dissolved back into water. Namely, during the 60–90 ns time frame of the MD simulation, the number of clusters specifically increased from 1 to 2–3 (Figure S4A). While in the system of Arg14 peptides, a higher number of clusters was caused not by peptide dissolvation but by lost interaction between HA polymers (Figure S4G).

During the MD simulation, the breakdown of pre-existing clusters (complexes) was also observed in systems containing 10-residue polyarginine peptides with low (1.67) and high (0.63) molar ratios (Figures S4C and S4E). The nature of complex decomposition within the MD with 10 polyarginine peptides varied depending on the molar ratio. In the case of a lower molar ratio (system 2HA25-8Arg10, 0.63), not all polyarginine peptides were able to achieve a favorable binding orientation due to their spatial distribution on the HA surface, and between 33 and 100 ns of the MD simulation, 1–2 individual polyarginine peptides dissociated from the pre-existing HA–polyarginine complex (Figure S4E). In systems with higher molar ratios (specifically, more HA units such as 2HA25-3Arg10), the observed increase in the number of clusters did not result in the detachment of free polyarginine molecules from the HA surface (Figure S4C, 30–60 ns) but due to the disruption of interactions between HA molecules. This phenomenon suggests that HA has a preference for interacting with the Arg peptide rather than with other HA molecules and also extended the time needed for complex formation and system equilibration.

Breakdown of the HA–polyarginine peptide complexes at lower molar ratios could contribute to the understanding of the specific first part of the ITC titration curve reported for the Arg10 peptide, where isotherm resembled the isotherms of shorter arginine oligomers.^14^

In addition, we could expect that at low HA concentrations with a small number of Arg peptides, separated HA molecules will not aggregate together. Polyarginine peptides played an integral role in the interaction with HA at molar ratios of around 1 and higher, leading to the formation of larger particles (Figure S2C,D).

Fluctuations of HA residues during the equilibrated period were determined by RMSF and are shown in Figures S1–S3 (bottom), Figure S5, and Figure 1C. The most flexible parts of HA are terminal HA units. The difference in flexibility of the nonterminal HA is determined by the interactions formed between the HA and the peptides. HA without polyarginine peptide has the highest flexibility at 10th–11th and 15th–16th units of HA; both are 10 units distant from the closest terminal peaks with the highest deviation from the linear conformation shown in Figure S5.

HA molecules in systems with long peptides (Arg12 and Arg14) changed their conformation from linear to bent more significantly—a higher RMSF with 2–3 distinct peaks for the central part of the HA molecule was observed for one of the HA polymers (Figure S2D–F). An increase in the number of peaks (to 3) and distribution of flexible regions (peaks) on the RMSF graph for HA molecules in the 2HA25-6Arg8 system were observed compared to the system without polyarginine peptides.

The RMSF graphs for HA with Arg10 were similar for HA alone (Figure S5B and Figure 1). A molar ratio of one with a peptide of 10 residues in size had a stabilizing effect on the structure of HA (lower RMSF in Figure S5B). A decrease in the peptide number (corresponding to the higher molar ratio, 1.66, in MD simulation with 3Arg10) did not change the flexibility of the HA polymer with respect to MD without peptides; the number of peaks and the total RMSF values were similar to the case with MD of HA polymers without peptides (Figure S5B). Generally, we can see that an increase in the number of Arg residues able to interact with the HA polymer leads to the restriction of HA flexibility (Figure S5B).

### Analysis of the Size, Shape, and Distribution of HA–Polyarginine Particles during MD Simulations

Changes in the organization of macroparticles formed by HA and polyarginine peptides were analyzed both visually and through calculations of RMSD, RMSF, the radius of gyration, and distances between terminal residues.

**Figure 2:**
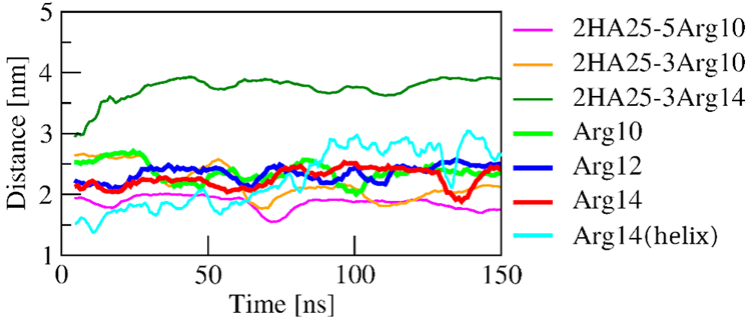
Distance between N- and C-terminals (calculated as the distance between C-alpha atoms of terminal Arg residues)/per amino acid residue in the MD simulation of Arg polypeptides made by 10, 12 and 14 amino acids in complexes with HA and without HA.

Polyarginine peptides containing 4, 8, 10, and 14 Arg residues without HA are solvated as extended peptides and preserved their conformation during MD simulation without the formation of regular structural elements (e.g., helices). Polyarginine peptide from 14 Arg residues with an initial helical structure lost regular secondary structure during the first 70 ns of MD and received an extended conformation. Distances between terminal amino acid residues of extended peptides were stable and did not significantly decrease during MD simulations (Figure 2).

During the MD simulation, the conformation of HA polymers exhibited an extended “wave-like” structure, similar to what has been reported in previous study,^29^ with a small linear decrease during MD simulation (Figure S2A). Although the possibility existed for separated HA polymers to interact with each other, we did not observe the formation of a single complex from two distinct HA25 molecules during the 150 ns duration of the MD simulation. In the MD simulations involving shorter hyaluronan molecules (4 HA units, data not shown), the HA polymers formed unstable complexes that rapidly dissociated.

**Figure 3:**
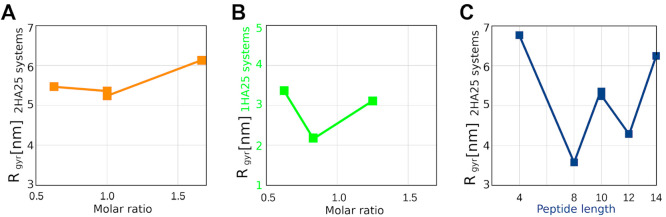
(A, B) Change in the radius of gyration with molar ratio: data for 10-residue peptides and 2HA25 (A); data for 10-residue peptides and 1HA25 (B). (C) Change in the radius of gyration with different peptide lengths.

In the presence of polyarginine peptides, the behavior of HA polymers exhibited a notable difference, as they were able to form stable complexes with polyarginine, termed HA–polyarginine complexes (Figures S1–S4, Figure 1). The resulting particles exhibited an irregular shape. Regions of HA that interacted with peptides underwent conformational changes, forming bends and loops. The linear size and shape of the HA–polyarginine peptide complex were dependent on the molar ratio, the length of the polyarginine peptide, and the length of the HA polymer (Figure 1A, Figure 3, and Table 1).

**Table 1:** Averaged Values of the Radius of Gyration and the Number of HBs

		*R*_gyration_, direction				number of HBs		
system	molar ratio	average	*x*	*y*	*z*	HA–water	HA–peptide	HA–Arg^a^
1HA25-4Arg10	1.25	3.11	2.83	2.53	2.14	318	33.6	1.68
1HA25-3Arg10	0.83	2.18	1.91	1.48	1.86	293	47.1	1.57
1HA25-2Arg10	0.625	3.37	2.9	2.87	2.28	284	45	1.13
2HA25-8Arg10	0.63	5.47	5.16	5.79	4.34	588	75	0.95
2HA25-5Arg10	1.00	5.36	4.8	4.45	3.8	636	62	1.24
2HA25-5Arg10, 100 mM NaCl	1.00	5.24	3.25	4.4	4.96	594	61	1.22
2HA25-12Arg4	1.04	6.77	1.1	6.72	6.72	568	83	1.73
2HA25-4Arg12	1.04	4.29	3.92	3.77	2.67	636	68	1.24
2HA25-6Arg8	1.04	3.58	3.01	3.13	2.57	603	69	1.44
2HA25-3Arg14	1.19	6.25	5.22	5.32	4.71	613	66	1.57
2HA25-3Arg10	1.67	6.13	3.6	5.4	5.7	656	43	1.43
2HA25		6.09	5.6	3.6	5.5	735	0	0

a Number of HB is calculated per a single Arg residue.

Small HA polymers composed of four HA units maintained their linear conformation when forming complexes with longer polyarginine peptides that covered the surface of HA (Figure S1B,C, very low RMSF and RMSD values). In the resulting particles, the charge ratio of HA/Arg was either 2/5 or 2/15. Therefore, the number of arginine residues adsorbed on the HA surface was not solely determined by charge ratio of the molecules, as was expected based on the precipitation;^14^ instead, it was closely related to the shape of the formed particle.

The modeling study could not directly address the impact of HA conformation (linear or rod-like versus bent) on high molecular weight HA, as discussed in the experimental work.^14^ This limitation arose from the relatively short length of the longest HA model that could be handled in the study.

The compactness of the formed complexes was evaluated by analyzing the radius of gyration (Figure 1D and Figure S6). At a molar ratio of approximately 1, the presence of short peptides (4 residues) resulted in the formation of linear complexes with bent HA, but without the formation of loops. Notably, the 2HA25-12Arg4 complex exhibited the highest radius of gyration among them (Table 1). HA–peptide complexes exhibited less compact particle formation also with a smaller number of 10-residue peptides (e.g., the 2HA25-3Arg10 complex in Figure S3A and Table 1) or with 14-residue polyarginine peptides (the 2HA25-3Arg14 complex in Figure S2E,F).

The shape of the particle with long (Arg14) peptides also depended on the initial orientation of the compounds. Arg10 formed the most compact particle at a molar ratio of 1 (Figure 1A,D).

Experimental evidence confirmed the saturation of HA–polyarginine peptide interaction sites at a molar ratio of approximately one.^14^ We anticipated that longer peptides would exhibit similar behavior to that of 2 HA molecules, potentially resulting in the aggregation of HA regions and the formation of larger particles. However, data from the MD simulation of 10-residue polyarginine peptides revealed that the saturation point occurred near a molar ratio of 1.6, which was higher than observed in the experiment for small weight HA. This difference could potentially be attributed to the higher concentration employed in the MD simulation and corresponds to the increase of saturation point observed for high molecular weight HA.^14^

**Figure 4:**
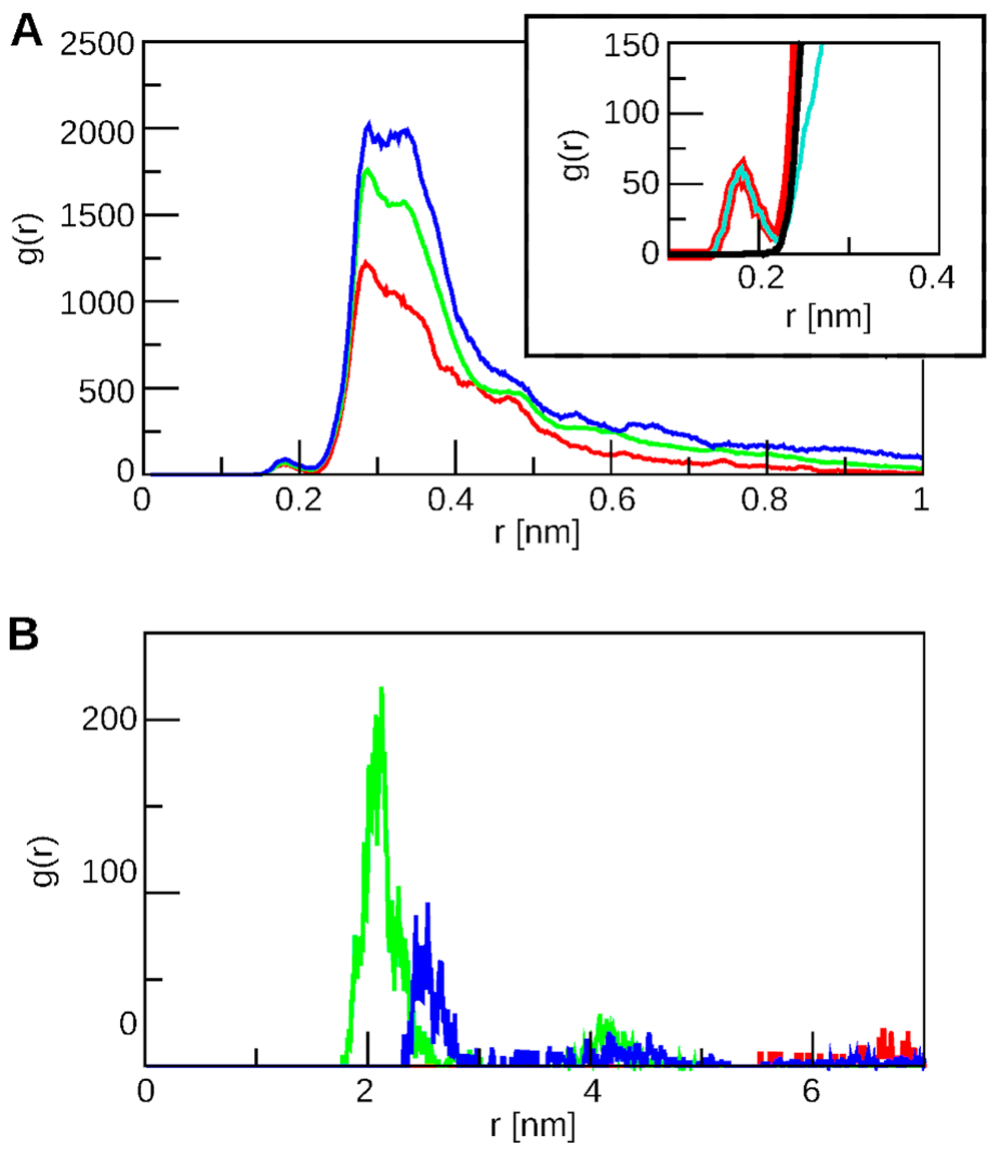
RDF in complexes with 1HA25 (for 180–200 ns of MD). Color scheme: red, 1HA25-2Arg10; green, 3HA25-3Arg10; blue, 1HA25-4Arg10. (A) Distribution of atoms of polyarginine peptides with respect to the HA surface. Inset represents the difference between the RDF of the main chain and side chain of the peptide in the 1HA25-2Arg10 system (black: side chain RDF; cyan: main chain; red: main and side chains RDF). (B) Distribution of polyarginine peptides with respect to each other, calculated for the centers of mass.

The organization of the formed HA–polyarginine peptide particles was assessed using RDF (radial distribution function). Polyarginine peptides formed only one layer with respect to the surface of HA molecules (Figure S8B and Figure 4). There is one clear sharp peak at 0.3 nm on the RDF of the Arg atoms with respect to the HA surface.

The distribution of one HA25 polymer with respect to the surface of another HA25 polymer also shows only one sharp peak at a distance of 0.327 nm in systems with 10-residue peptides (2HA25-5Arg10 (run2), 2HA25-5Arg10 (100 mM NaCl)). However, an exception was found in the case of 2HA25-3Arg10, where a high peak was not observed. This indicates that the majority of HA atoms from one polymer were arranged in a single layer in relation to the other polymer.

The RDF of HA molecules in the 2HA25-12Arg4 system has a plateau at a distance of 0.2–0.6 nm with three smaller peaks (Figure S8A left, red line). The highest peak in the RDF of polyarginine peptides with respect to the HA surface is at a distance of 0.38 nm (Figure S8B left, red line); hence, we concluded that HA molecules in this system had a linear conformation and were separated by a polyarginine layer. In the systems 2HA25-6Arg8, 2HA25-4Arg12, and 2HA25-3Arg14 (run1), circle-like multilayer particles were formed (Figures S2 and S3). A first low RDF peak observed in the distribution of polyarginine peptides with respect to the HA surface (Figure 4A, inset) corresponds to the main chain atoms of the peptide and shows that they were closer to the HA surface than to the side chain.

The first peak in the RDF of the center of mass of polyarginine peptides corresponding to 2.1 nm shows that they are distributed at a distance longer than required for HB formation (Figure S8C and Figure 4B).

It is worth mentioning that the difference in the terminal effects of polyarginine–HA binding is not significant. There is no clear preference for the polyarginine peptide to bind to the terminal or central part of the HA polymer (from the RDF calculation, data not shown); however, it should be noticed from the trajectory analysis that binding at the terminals and the internal part of the HA molecule leads to different conformational changes (Figures S1–S3, Figure 1).

### Analysis of the Interactions Formed between Different Components of the Systems (HA, Polyarginine Peptides, Water, and Ions) during MD Simulation

HA molecules in the presence of polyarginine peptides form 5–20 internal hydrogen bonds (stable MD period). Interaction between polyarginine peptides and Arg residues was not determined in MD simulations without HA (Figures S1 and S8C). This could be explained by the nature of Arg residues, which form peptides with positive charges on the surface.

There are two primary types of interactions formed by Arg residues within the same peptide: hydrogen bonds established between the guanidino group of the side-chain and the main-chain oxygen (Figure S7A,B) as well as the stacking interaction of guanidino groups (Figure S7A). Guanidino group stacking is described in the literature and is called cation ion-pairing.^30^ Similar interactions are also found between guanidino groups of polyarginine peptides adsorbed at the HA surface. Cation ion-pairing interactions were not observed in MD simulations with shorter Arg4 peptides. Formation of these interactions by Arg10 (decamer) explains a specific behavior in the ITC experiments, where the reaction isotherm did not have a typical sigmoidal shape as described in our previous work.^14^

The maximum possible number of internal HBs formed by polyarginine peptides built from 4, 10, 12, and 14 residues was 2, 4, 5, and 7, respectively. The most frequently formed HB was related to hydrogen bond interactions between Arg residues, separated by one Arg residue (the HB between *n* and *n* + 2 residues, Figure S7C). The presence of HA in the simulation caused a modification in the internal hydrogen bonding interaction pattern between Arg residues in peptides. In addition to the existing HBs, new HBs between Arg residues separated by 2, 3, and 4 residues were observed. The frequency of HB interactions between polyarginine peptides in complex with HA decrease compared to systems with peptides alone. The most significant frequency decrease in MD with Arg4, Arg12, and Arg14 Arg was more than 2 times. The number of internal HBs between Arg residues in peptides in complexes with HA decreased during MD simulations with Arg10 and Arg12. During the molecular dynamics simulation of Arg14 with HA, the number of interactions either decreased (cyan line in Figure S7D) or remained similar to the initial state of the unbound peptide (green line in Figure S7D).

**Figure 5:**
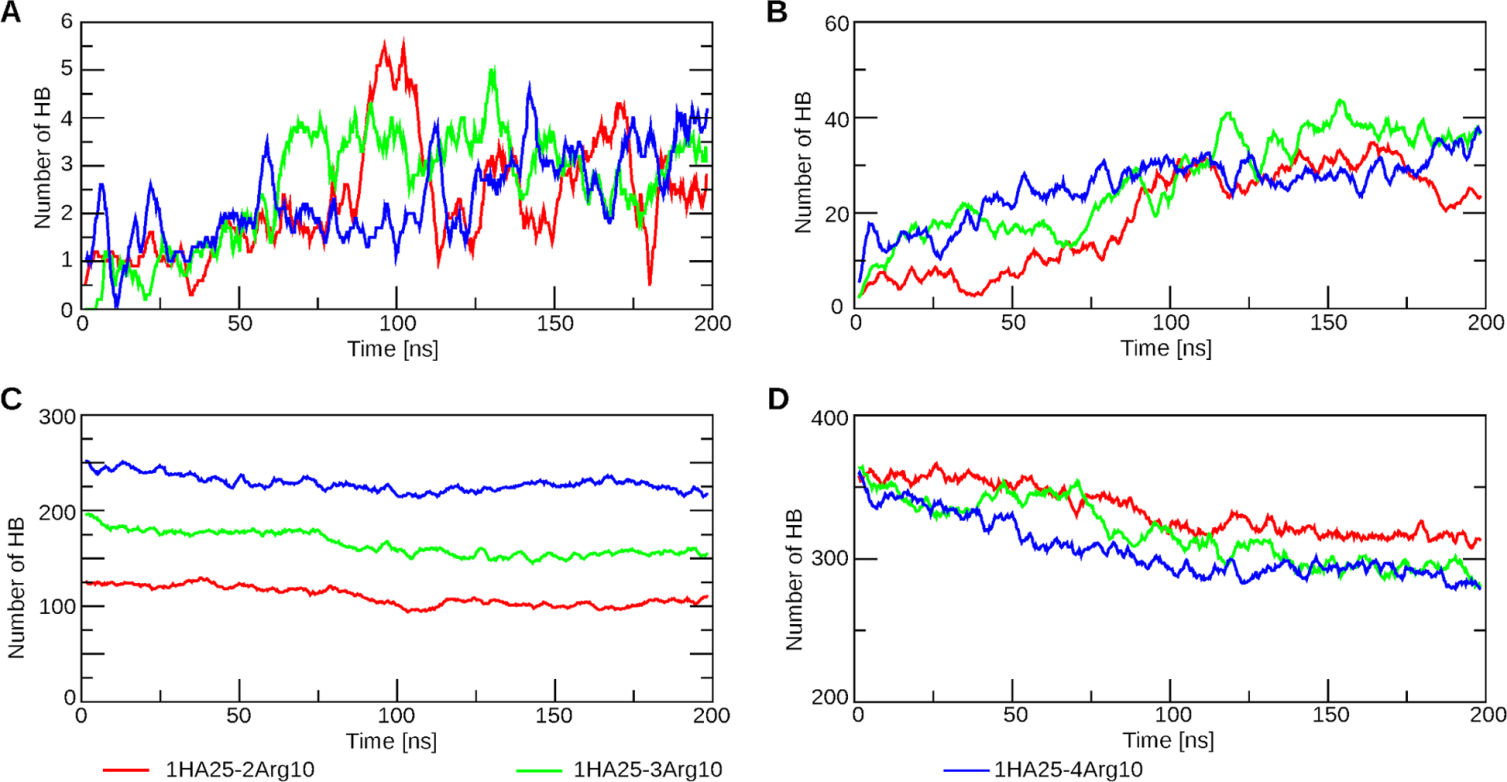
Number of HBs in the systems with 1HA25 polymer formed during MD. Data from MD are averaged over 10 snapshots. Legend is common for all graphs and is shown below. (A) HA–peptides (main chain); (B) HA–peptides (side chain); (C) polyarginine peptides–water; (D) HA–water.

**Figure 6:**
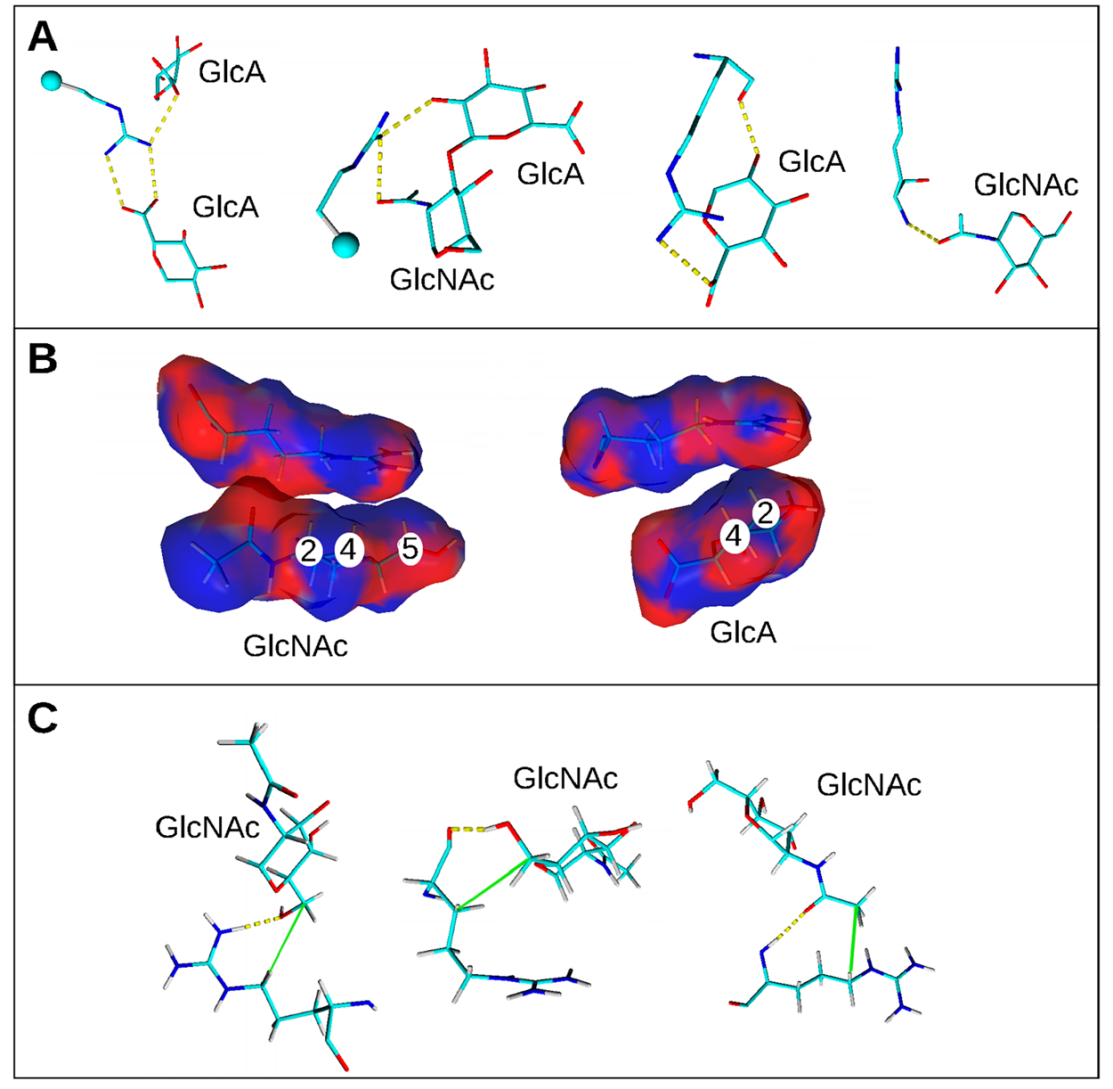
Interactions between Arg residues and HA. Snapshots from the MD of 2HA25-5Arg10 after 100 ns were used for representation. (A) Hydrogen bonds; HBs are shown by yellow dotted lines, and hydrogens are hidden. (B) Stacking interaction; residues are colored by electrostatic potential surfaces made by the Poisson–Boltzmann method: red color, negative charge; blue color, positive charge. Carbohydrate atoms of HA, participating in stacking, are labeled. (C) Hydrophobic interactions. Atoms, forming hydrophobic interactions, are connected by green lines, and HBs are shown by yellow dotted lines.

Polyarginine peptides and HA interact by means of HB formation (including salt bridges as a specific type of HB), hydrophobic interaction, and stacking interactions (Figures 5 and 6). Electrostatic interactions (which include HBs) play an important role in the binding. The HBs formed between HA and Arg involve hydroxyl groups of carbohydrate molecules of HA and NH_3_^+^, NH^2^, NH, and COO^–^ of Arg. Strong salt bridges can be established by the carboxyl of GlcA and the guanidine group of the Arg side chain (Figure 6A).

The majority of HBs are established between HA and the side chains of Arg (see Figure 5). Interactions between HA and the main chain are possible at the peptide terminals or when the Arg polypeptide undergoes a conformational change from a linear to a bent structure. Shorter residues exhibit a greater number of main-chain hydrogen bond (HB) interactions, as they have more exposed N-terminals that can interact with HA. Additionally, shorter residues tend to form more HBs per Arg residue compared to longer ones (Table S2).

During complex formation, the number of HBs among HA–HA, HA–polyarginine peptides, and different Arg peptides was increased (Figure S9A–E and Figure 5A,B). HBs formed between HA and Arg substituted interactions with water molecules (Figure S9F,G and Figure 5C,D).

Stacking interactions between HA and Arg are determined by the presence of apolar hydrophobic surfaces on HA units (GlcA and GlcNAc), formed by carbon- or nitrogen-bound hydrogens.^31^ Carbohydrate–amino acid stacking interactions are well-studied in the context of aromatic residues^32^ but are not well described for Arg residues. Arg residues form stacking interactions with three possible carbohydrate surfaces: with two apolar sides of GlcNAc (one is formed by hydrogens at C1, C3, and *N*-acetyl group; the other is formed by hydrogens at C2, C4, and C5) and with one apolar surface of GlcA formed by hydrogens at C2 and C4) (Figure 6B).

In addition to HBs and stacking interactions, hydrophobic contacts influence the orientation of Arg residues with respect to HA units, which are formed mainly by GlcNAc residue (CH_2_ at the C5 atom or CH_3_ at the *N*-acetyl group) and Arg (the CH_2_ group) (Figure 6C).

During the MD simulation, we observed that the orientation of Arg residues necessary for the formation of stacking and hydrophobic interactions with HA is stabilized by hydrogen bonds formed by neighboring Arg residues in peptides. The stabilization of residue interactions cannot be achieved solely by the interaction between HA and a single Arg residue. Therefore, the length of the polyarginine peptide plays a crucial role in enhancing the binding between HA and the polyarginine peptide by stabilizing the orientation of Arg side chains for the formation of these contacts. Longer peptides facilitate the additional formation of stacking and hydrophobic interactions with HA with respect to short peptides (1–4 Arg), further enhancing the binding.

Polyarginine peptides and HA form many HBs with water molecules (Figure S9F,G and Figure 5). The total number of HBs formed between polyarginine peptide/HA and water decreased during MD simulations and depended on the length of the peptide and the number of peptides. As the number of Arg residues in the system increased, the number of hydrogen bonds with water also increased (Table S2).

The data presented in Table S2 indicate that shorter polyarginine peptides in complexes with HA (systems 2HA25–12Arg4 and 2HA25–6Arg8) form more HBs with water than longer peptides (2HA25–3Arg14) at the same molar ratio. Arg peptides in 2HA25–3Arg14 systems formed the smallest number of HBs with water upon adsorption on the HA surface. Therefore, it is expected that the desolvation of smaller peptides will be less energetically favorable compared with longer peptides. This difference in desolvation energy likely contributes to the varying abilities of polyarginine peptides of different lengths to form stable complexes with HA. From the previous experiments^14^ was concluded that with the increase of molar ratio for decamer hydration shell lost waters. In MD simulations of 1HA25 with different molar ratios, we observe a lower number of HB formed by water and peptide at higher molar ratio, but not HA (Table S2).

The final number of HBs formed between HA–polyarginine peptide complexes and water during the equilibrated period was inversely proportional to the molar ratio (Figure S9H). In the molar ratio range 1.2–1.67, we observed an increase in the number of hydrogen bonds (HBs). This finding aligns with the experimentally observed saturation of HA–polyarginine complexes at a molar ratio of 1.^14^ Complexes with peptides of different lengths and a molar ratio of around one showed that the number of HBs between HA and Arg had a linear relation (Figure S9I).

### Analysis of the Shape, Size, and Organization of HA–Polyarginine Complexes at Higher Salt Concentrations

The sizes of the HA–polyarginine peptide complexes at different salt concentrations of NaCl were comparable (Table 1). However, certain differences are noticeable, such as the positioning of polyarginine peptides at the surface of HA (Figure S3D) and the number of hydrogen bonds (HBs) formed between HA polymers during the MD simulations. At a higher ionic strength, more HBs were formed between HA polymers (Figure S10D). This was not an artifact of the initial configuration because, at the beginning of MD simulations, HA polymers did not interact. Moreover, at higher NaCl concentrations HA polymers lost surface water and formed less HBs (Figure S10B). It can be stated that molecules that remove water from the surface of HA such as peptides or ions can improve the aggregation of HA molecules.

Sodium ions are attracted to HA molecules; however, this interaction appeared to be short-lived as the distribution of ions on the HA surface resembled that of the surrounding solvent, as depicted by the lower first peak in Figure S10F. The attraction of Cl anions to Arg was more significant according to the higher first peak in the RDF of Cl with respect to Arg (Figure S10E). This indicates that the concentration of Cl ions surrounding the peptides was higher compared to the bulk solution, enabling the formation of a negatively charged layer. This layer, in turn, hindered the attraction of Arg to the HA surface (as shown in Figure S4D), confirming experimentally determined weaker interaction between HA and Arg,^14^ and ultimately resulted in the formation of linearly extended complexes between HA and polyarginine (Figure S3D).

### Analysis of the Free Energy of Binding of Polyarginine Peptides to HA during MD Simulations

**Figure 7:**
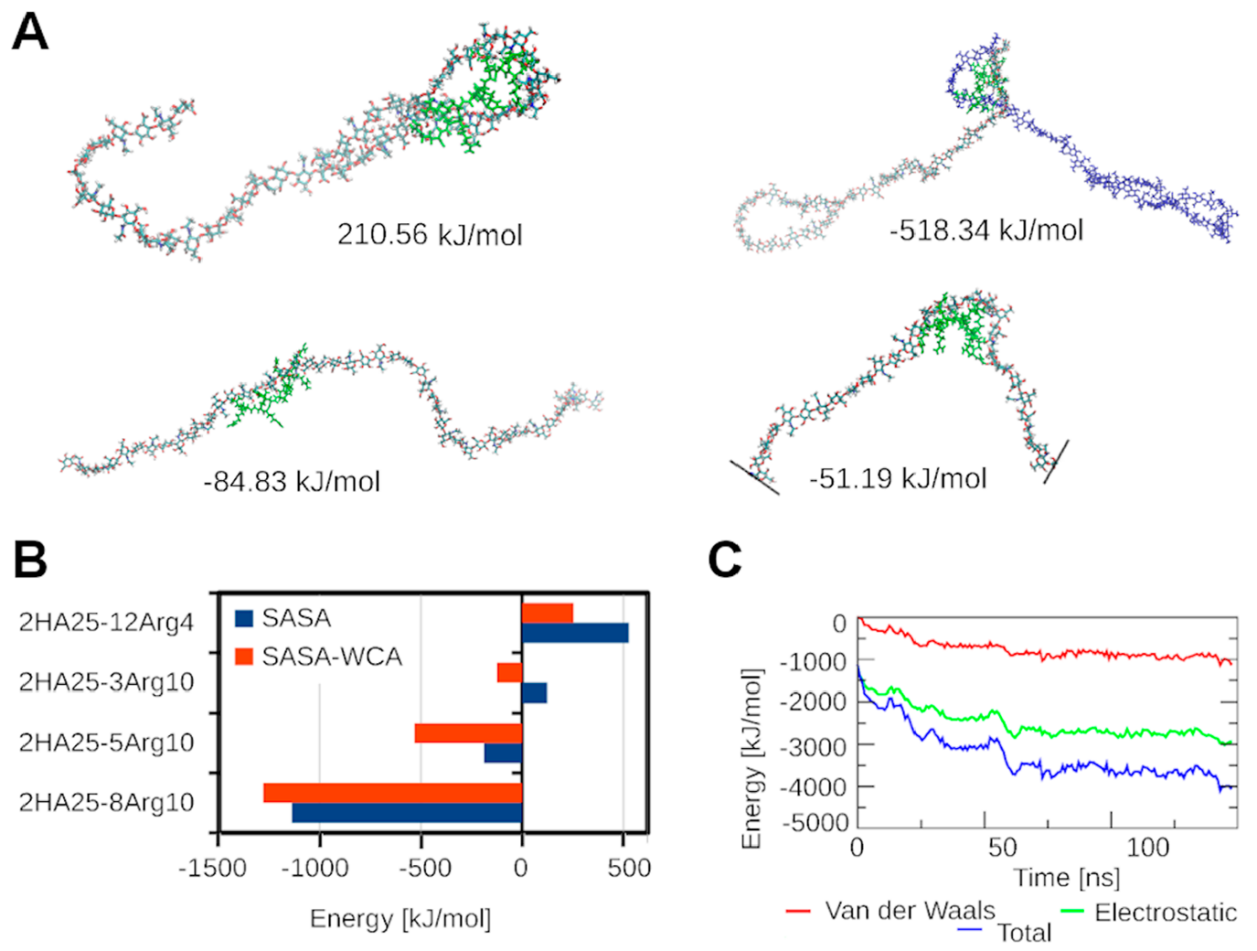
Analysis of the free energy of binding. (A) Representation of Arg10 binding modes with respect to the HA: at the terminal loop, at the terminal between 2HA, with linear conformation, and with bent conformation with corresponding free energies of binding. In one, HA molecule, the elements are colored differently; the other HA molecule is blue. Polyarginine peptide is green. (B) Free energy of polyarginine peptides binding for different complexes. Results are compared for different models of the apolar solvation energy calculation: SASA and SASA-WCA. (C) Time evolution of molecular mechanics components of free energy of binding shown for the system 2HA25–3Arg10.

The free energy of binding was estimated by the MM-PBSA method, as described in the Materials and Methods section. The results of the energy calculation are summarized in Figure 7 and Tables S3–S4. The MM-PBSA method in GROMACS does not include entropic terms; therefore, the calculated free energy of binding is proportional to the enthalpy of binding.

The interactions between HA polymers with different numbers of polyarginine peptides were analyzed and are illustrated in Figure 7. The effect of different ionic strengths on the free energy of binding was analyzed for the 2HA25–5Arg10 complex by changing the value of the solvent dielectric constant. The results are summarized in Table S4.

The calculated total free energy of binding exhibited positive values and proved to be sensitive to the method employed for apolar energy calculation, namely, SASA, WCA, or SASA–WCA, as shown in Table S3. Apolar energy is directly related to SASA and signifies the energy needed for the formation of a cavity within the solvent. This aspect is incorporated into the SASA model. The attractive van der Waals interaction between the solvent and solute is accounted for in the WCA model. To encompass both aspects of solvation energy, the SASA–WCA model provides a comprehensive representation. Despite the fact that the apolar energies calculated by different methods were not the same, the general difference in energy between different systems was preserved (Table S3 and Figure 7B).

The free energy of binding was higher for systems with shorter peptides (4 residues long). Complexes with similar lengths of peptides (10-residue peptides) showed an improvement in binding (a lower free energy of binding) with a decrease in the molar ratio (the best was for 2HA25–8Arg10). The electrostatic component of the free energy of binding (which also accounts for HBs) made the highest contribution to the total energy; hence, it played an important role in the stabilization of HA–polyarginine peptide complexes (Figure 7C and Table S3).

The influence of the mutual orientation of the HA polymer and peptide on the free energy of binding was analyzed for individual peptides according to the MD simulation of the selected snapshots from the MD of the 2HA25–3Arg10 system. The binding orientation of HA to a peptide is described on the basis of the change in conformation of the HA polymer (from the left to the right in Figure 7A): terminal loop, terminal between two HA, linear, and bent. The free energy of binding inside the terminal HA loop is much higher, and the formation of this complex is less energetically favorable. The most favorable free energy of binding was found for binding between two HA molecules due to favorable electrostatic interactions between HA and polyarginine peptide and due to the lower polar contribution to the solvation energy (Table S3).

Free energies of binding calculated confirmed the conclusion from experiments^14^ that polyarginine peptides did not show a preference for terminal binding. In addition, the free energy of binding calculations showed that the main drivers of binding include electrostatic and hydrophobic forces and additionally the potential energy of HA molecules (the conformational penalty).

Positive values of the free energy of binding reflect the fact that entropic cost is not included in this calculation, but from experimental data it should improve binding scores; another reason is a strong dependency of solvation energy on selected model and on dielectric constant of solute.

The results showed that the binding modes correlated to the free energy of binding. The binding of polyarginine peptide to the bent HA conformation is slightly less energetically favorable than binding to the same place in the extended mode. The bending of the HA polymer requires an extra energy input, which can be mitigated by the establishment of novel interactions with polyarginine. These interactions serve to offset the penalty associated with the conformational strain. This also means that interactions are more favorable for systems made with a smaller molecular weight of HA.

## Conclusions

The MD simulation validated the primary experimental findings, as stated in ref (14), to the interactions between HA and polyarginine peptides and the conformations of the formed complexes: improvement of interaction between HA at high salt concentration as an effect of loss of water shell, lack of the stable interaction between HA and Arg residues alone, and the important role of electrostatic interactions for Arg–HA binding.

However, in MD simulation, we observed that HA molecules absorbed more polyarginine peptides on the surface than expected by experimental precipitation molar ratio (equal to 1). Also, interpretation of the free energy of binding is not straightforward for these systems. Despite the relationship between different energies confirming worse binding for small peptides, absolute values depend very much on selected parameters (solvation model and dielectric constant).

Despite all-atom MD simulation allowed analysis of formed interactions and binding modes of polyarginine peptides to HA, a coarse-grained approach could be used for longer simulation to investigate full conformational space of HA–polyarginine particles. Neither HA nor polyarginine peptides form regular secondary structures, whether in water or in complexes. Additionally, HA molecules exhibit a low affinity to interact with each other in a solvent without the presence of added polyarginine. This is primarily due to the significant energy required for HA desolvation and the associated strain penalty. However, the addition of ions (such as NaCl) can effectively displace water molecules from HA, thereby enhancing the interaction between the HA polymers.

The likelihood of a polyarginine peptide being attracted from solvent to a specific region of HA is comparable for both the terminus and central portions, although there is more favorable energy for binding to the central region. Both monomers and peptides of Arg residues can interact with HA polymers; however, the stable complexes are formed only with peptides that are longer than four residues. The length of the peptide determines the linear size of the HA–polyarginine peptide complex. Polyarginine peptides consisting of more than 8 Arg residues induce a significant bend in the HA molecule. On the other hand, smaller (4 residue) polyarginine peptides exhibit weaker binding to HA and form linear-extended complexes.

The main driving forces behind complex formation are the electrostatic energy (including HBs), polar solvation energy, and the potential energy of HA. Energy calculations demonstrated a more favorable free energy of binding in systems with 10-residue peptides compared to those with 4-residue peptides. This can be attributed to the more favorable solvation polar energy observed in systems with 10-residue peptides, while the electrostatic contribution per Arg residue remained comparable between the two.

## Supporting Information Available

Tables with summary of modeled systems, formed HBs, free energy of binding for different complexes and ionic strength; figures with shapes of formed particles, number of clusters formed during MD, RMSF of HA units of HA25 molecules, radii of gyration, RDF calculation, analysis of HBs (PDF)

## Abbreviations

GlcA: d-glucuronic acid
GlcNAc: *N*-acetyl-d-glucosamine
HA: hyaluronic acid (hyaluronan; for polymers, the number of HA units is written after the letters, the number of polymers before: 2HA25-2 hyaluronic acid polymers made of 25 HA units)
HA unit: basic disaccharide unit of HA (formed by GlcA and GlcNAc)
5Arg10: abbreviation used for description of polyarginine peptides, the number of arginine residues in the peptide is labeled after “Arg”, the number of peptides before “Arg”
HB: hydrogen bond
MD: molecular dynamics simulation
RMSD: root-mean-square deviation
RMSF: root-mean-square fluctuation
SASA: solvent accessible surface
WCA: Weeks–Chandler–Andersen
RDF: radial distribution function.
