# Supplementary material for: Computational Study of Complex Formation between Hyaluronan Polymers and Polyarginine Peptides at Various Ratios
Source: Langmuir. 2023 Sep 29;39(40):14212–22. doi: 10.1021/acs.langmuir.3c01318 (PMC10569091; doi:10.1021/acs.langmuir.3c01318)
Supplement: Supplementary file 1 — la3c01318_si_001.pdf [file la3c01318_si_001.pdf]

Supporting Information

Computational study of complex formation between hyaluronan polymers and polyarginine peptides at various ratios

Natalia Kulik[a,*], *Babak Minofar*[b,*], *Adam Jugl*[c], *Miloslav Pekař*[c]

[a] Laboratory of Photosynthesis, Institute of Microbiology of the Czech Academy of Sciences, Novohradská 237 - Opatovický mlýn, 379 01 Třebon, Czech Republic, kulik@nh.cas.cz (N.K.)

[b] Faculty of Science, University of South Bohemia, Branišovská 1760, 37005 České Budějovice, Czech Republic, babakminoofar@gmail.com (B.M.)

[c] Faculty of Chemistry, Brno University of Technology, Purkyňova 118, 612 00 Brno, Czech Republic, xcjugl@fch.vut.cz (A. J.), pekar@fch.vut.cz (M.P.)

[*] Corresponding authors kulik@nh.cas.cz, babakminoofar@gmail.com

ABBREVIATIONS

GlcA, D-glucuronic acid; GlcNAc, *N*-acetyl-D-glucosamine; HA, hyaluronic acid (hyaluronan; for polymers, the number of HA units is written after the letters, the number of polymers before: 2HA25 – 2 hyaluronic acid polymers made of 25 HA units); HA unit, basic disaccharide unit of HA (formed by GlcA and GlcNAc); 5Arg10, abbreviation used for description of polyarginine peptides, the number of arginine residues in the peptide is labeled after 'Arg', the number of peptides before 'Arg'; HB, hydrogen bond; MD, molecular dynamics simulation; RMSD, root means square deviation; RMSF, root means square fluctuation; SASA, solvent accessible surface; WCA, Weeks-Chandler-Andersen; RDF, radial distribution function.

Table of Contents

Table S1. Summary of modelled systems S3
Table S2. Summary of HBs formed during MD S5
Table S3. Free energy of binding for different complexes S6
Table S4. Free energy of binding at different ionic strength S7
Fig. S1. Shapes of particles formed in small systems S8
Fig. S2. Shapes of particles S9
Fig. S3. Shapes of particles of the systems with Arg10 ....S10
Fig. S4. Number of clusters formed during MD for of polyarginine peptides-HA complexes with 2HA25 ....S11
Fig. S5. RMSF of HA units of HA25 molecules S12
Fig. S6. The radii of gyration of HA-polyarginine peptide complexes....S13
Fig. S7. Analysis of interactions between Arginines S14
Fig. S8. RDF calculation S15
Fig. S9. Analysis of HB in HA-polyarginine systems S16
Fig. S10.Number of HBs at different concentrations of NaCl S18
References S19

Table S1. Summary of modelled systems

	Solute content	Short name for the system	MD length/stability time *	Molar ratio **
1	**Arginine without HA**			
1.1	Arg residue, total 10 amino acids in the system	10Arg1	50 ns/ 40-50 ns	
1.2	1 polyarginine peptide made from 4 Arg residues	1Arg4	50 ns/ 40-50 ns	
1.3	1 polyarginine peptides made from 10 Arg residues	1Arg10	150 ns/ 40-150 ns	
1.4	1 polyarginine peptides made from 12 Arg residues	1Arg12	150 ns/ 40-150 ns	
1.5	1 polyarginine peptides made from 14 Arg residues (extended structure)	1Arg14	150 ns/ 40-150 ns	
1.6	1 polyarginine peptides made from 14 Arg residues (helical structure)	1Arg14h	150ns/70-150 ns	
2	**HA without Arg**			
2.1	2 molecules of HA, each from 25 HA units; HA molecules are separated in a space at the beginning of MD	2HA25	150 ns/ 80-150 ns	
3	**HA and Arg**			
3.1	1 molecule of HA from 4 units, 10 Arg residues	1HA4-10Arg1	250 ns/50-250 ns	0.4
3.2	1 molecule of HA from 4 units, 1 polyarginine peptide from 10 amino acid residues	1HA4-1Arg10	250 ns/50-250 ns	0.4
3.3	2 molecules of HA, each from 4 units, 6 polyarginine peptides made from 10 residues	2HA4-6Arg10	100ns /60-100 ns	0.13
3.4	1 molecule of HA from 25 units, 2 polyarginine peptides made from 10 residues	1HA25-2Arg10	200ns/ 120-200 ns	1.25
3.5	1 molecule of HA from 25 units, 3 polyarginine peptides made from 10 residues	1HA25-3Arg10	200ns/ 140-200 ns	0.83
3.6	1 molecule of HA from 25 units, 4 polyarginine peptides made from 10 residues	1HA25-4Arg10	200ns/ 180-200 ns	0.63
3.7	2 molecules of HA, each from 25 units, 12 polyarginine peptides made from 4 residues	2HA25-12Arg4	150 ns /100-150ns	1.04
3.8	2 molecules of HA, each from 25 units, 6 polyarginine peptides from 8 residues	2HA25-6Arg8	110 ns /80-110ns	1.04
3.9	2 molecules of HA, each from 25 units, 3 polyarginine peptides from 10 residues	2HA25-3Arg10	150 ns/100-150 ns	1.67
3.10	2 molecules of HA, each from 25 units, 5	2HA25-5Arg10,	60 ns/50-60ns	1

	polyarginine peptides made from 10 residues, run1, 0.01 mM NaCl	run1		
3.11	2 molecules of HA, each from 25 units, 5 polyarginine peptides from 10 residues, run2, 0.01 mM NaCl	2HA25-5Arg10, run2	200 ns/80-120 ns	1
3.12	2 molecules of HA, each from 25 units, 5 polyarginine peptides from 10 residues, 100mM NaCl	2HA25-5Arg10, 100mM NaCl	150 ns/100-130 ns	1
3.13	2 molecules of HA, each from 25 units, 8 polyarginine peptides from 10 residues	2HA25-8Arg10	150 ns/100-150 ns	0.63
3.14	2 molecules of HA, each from 25 units, 4 polyarginine peptides from 12 residues	2HA25-4 Arg12	108 ns/80-108 ns	1.04
3.15	2 molecules of HA, each from 25 units, 3 polyarginine peptides from 14 residues, run1	2HA25-3 Arg14,run1	200 ns/100-200 ns	1.19
3.16	2 molecules of HA, each from 25 units, 3 polyarginine peptides from 14 residues, run2	2HA25-3 Arg14, run2	95 ns/87-95 ns	1.19

* The stability time was selected on the basis of the stable RMSD of HA and the time from which a stable cluster was formed (in most HA-Arg systems is 1). This period corresponds to the analyzed MD time for the calculation of averaged values and RDF values.

** The molar ratio is the number of HA units divided by the number of Arg residues as introduced in reference [1].

Table S2. Summary of HBs formed during MD

Maximum values in columns for 2HA25 polymer are highlighted in ***bold and italics***, minimum in _underlined italics_. Number of hydrogen bonds is calculated for equilibrated period of MD.

Molar ratio [1]	Short name for system	Number of HBs					
		Pept.-HA, total	Pept.-HA, normalized*	HA-water	Pept.-water, total	Pept.-water, normalized*	System – water, total**
1.25	1HA25-2Arg10	33.6	1.68	318	103	5.15	421
0.83	1HA25-3Arg10	47.1	1.57	293	154	5.13	447
0.63	1HA25-4Arg10	45	1.13	284	221	5.52	505
1.04	2HA25-12Arg4	***83***	***1.73***	568	286	***5.95***	854
1.04	2HA25-6Arg8	69	1.44	603	260	5.41	863
1.67	2HA25-3Arg10	_43_	1.43	***656***	_155_	5.17	811
1	2HA25-5Arg10, run1	62	1.24	636	277	5.54	913
1	2HA25-5Arg10, run2	74	1.48	_557_	248	4.96	_805_
1	2HA25-5Arg10, 100mM NaCl	61	1.22	594	269	5.38	863
0.63	2HA25-8Arg10	75	_0.94_	588	***439***	5.49	***1027***
1.04	2HA25-4Arg12	68	1.41	636	249	5.19	885
1.19	2HA25-3Arg14,run1	66	1.57	613	205	4.88	818
1.19	2HA25-3Arg14, run2	64	1.52	650	199	_4.74_	849
0.4	1HA4-10Arg1	2.4	0.2	59	83.1	8.3	142.1
-	1Arg10	0	0	0	63	6.3	-
-	1Arg12	0	0	0	81	6.75	-
-	2HA25	0	0	735	0	0	-

* Normalization is performed to the number of Arg residues.

** Total number of HBs formed by the system HA-Arg is the sum of HBs for Arg-water and HBs for HA-water.

Table S3. Free energy of binding for different complexes

Model for apolar energy calculation 2	Van der Waals energy, kJ/mol	Electrostatic energy	Polar solvation energy	Apolar solvation - SASA model	Apolar solvation - SASA-WCA model	Binding energy
**2HA25-3Arg10, (t=90-100 ns), interaction of 2HA25 and 3 Arg10**						
SASA-only	-847.57	-2684.18	3836.4	-121.69	-	182.96
WCA-only	-//-	-//-	-//-	0	560.43	865.08
SASA-WCA	-//-	-//-	-//-	-1214.64	727.57	-182.47
**2HA25-3Arg10, interaction of 1HA25 and one polyarginine peptide in terminal HA loop**						
SASA-only	-317.08	-768.35	1557.47	-49.35	-	422.7
SASA-WCA	-//-	-//-	-//-	-545.46	283.97	210.56
**2HA25-3Arg10, interaction of 2HA25 and one polyarginine peptide bound between HA polymers**						
SASA-only	-301.9	-1020.83	1393.2	-50.06	-	84.39
SASA-WCA	-//-	-//-	-//-	-849.52	260.7	-518.34
**2HA25-3Arg10, interaction of 1HA25 and one polyarginine peptide bound to linear HA**						
SASA-only	-225.638	-562.454	973.625	-32.968	-	152.563
SASA-WCA	-//-	-//-	-//-	-459.895	183.535	-84.827
**2HA25-3Arg10, interaction of 1HA25 and one polyarginine peptide bound to bent HA**						
SASA-only	-261.881	-615.898	1111.94	-36.008	-	198.154
SASA-WCA	-//-	-//-	-//-	-495.472	210.116	-51.194
**2HA25-5Arg10, (t=50-60 ns), interaction of 2HA25 and all polyarginine peptides**						
SASA-only	-1319.11	-4518	5695.355	-184.485	-	-236.877
SASA-WCA	-//-	-//-	-//-	-1514.093	1128.142	-528.343
**2HA25-8Arg10, run2, (t=90-100 ns), interaction of 2HA25 and all polyarginine peptides**						
SASA-only	-1777.777	-7124.556	7986.181	-233.972	-	-1150.128
SASA-WCA	-//-	-//-	-//-	-1852.981	1499.116	-1270.019
**2HA25-12Arg4, (t=90-100 ns), interaction of 2HA25 and all polyarginine peptides**						
SASA-only	-1382.22	-4623.24	6725.37	-195.99	-	521.92
SASA-WCA	-//-	-//-	-//-	-1645.29	1217.65	290.26

Table S4. Free energy of binding at different ionic strength

Data are calculated for HA and polyarginine peptides in the system 2HA25-5Arg10 (run 1), calculated for different ionic strengths (modelled as different solvent dielectric constants). Only the SASA model is used for solvent energy calculation.

Solvent dielectric constant	Van der Waals energy	Electrostatic energy	Polar solvation energy	Apolar energy	Binding energy
20	-1153.211	-5055.625	5714.65	-164.59	-625.79
30	-//-	-//-	5838.08	-//-	-535.35
40	-//-	-//-	5906.51	-//-	-466.91
50	-//-	-//-	5951.56	-//-	-421.86
60	-//-	-//-	5984.22	-//-	-389.22
80	-//-	-//-	6029.572	-//-	-343.857

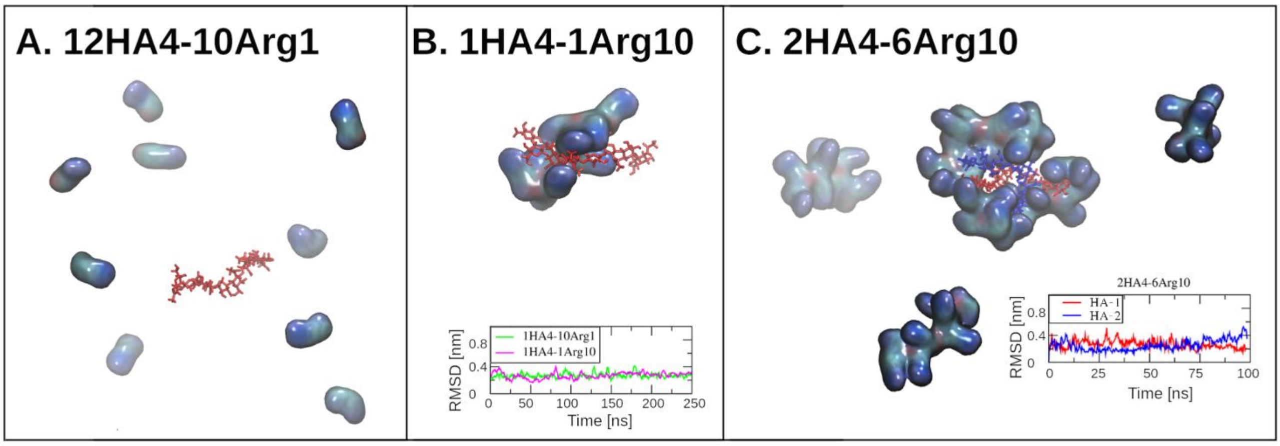

Fig. S1. Shapes of particles formed in small systems

A-C. Shapes are shown as formed at the end of the MD for the corresponding systems.

One HA polymer in the system is colored red (HA-1), the other HA polymer is colored blue (HA-2); polyarginine peptides are represented by surface representation and colored according to the atom name. The RMSD of HA molecules are shown under each representation. The RMSD of HA molecules for 1HA4-10Arg1 and 1HA4-1Arg10 are merged in B (bottom).

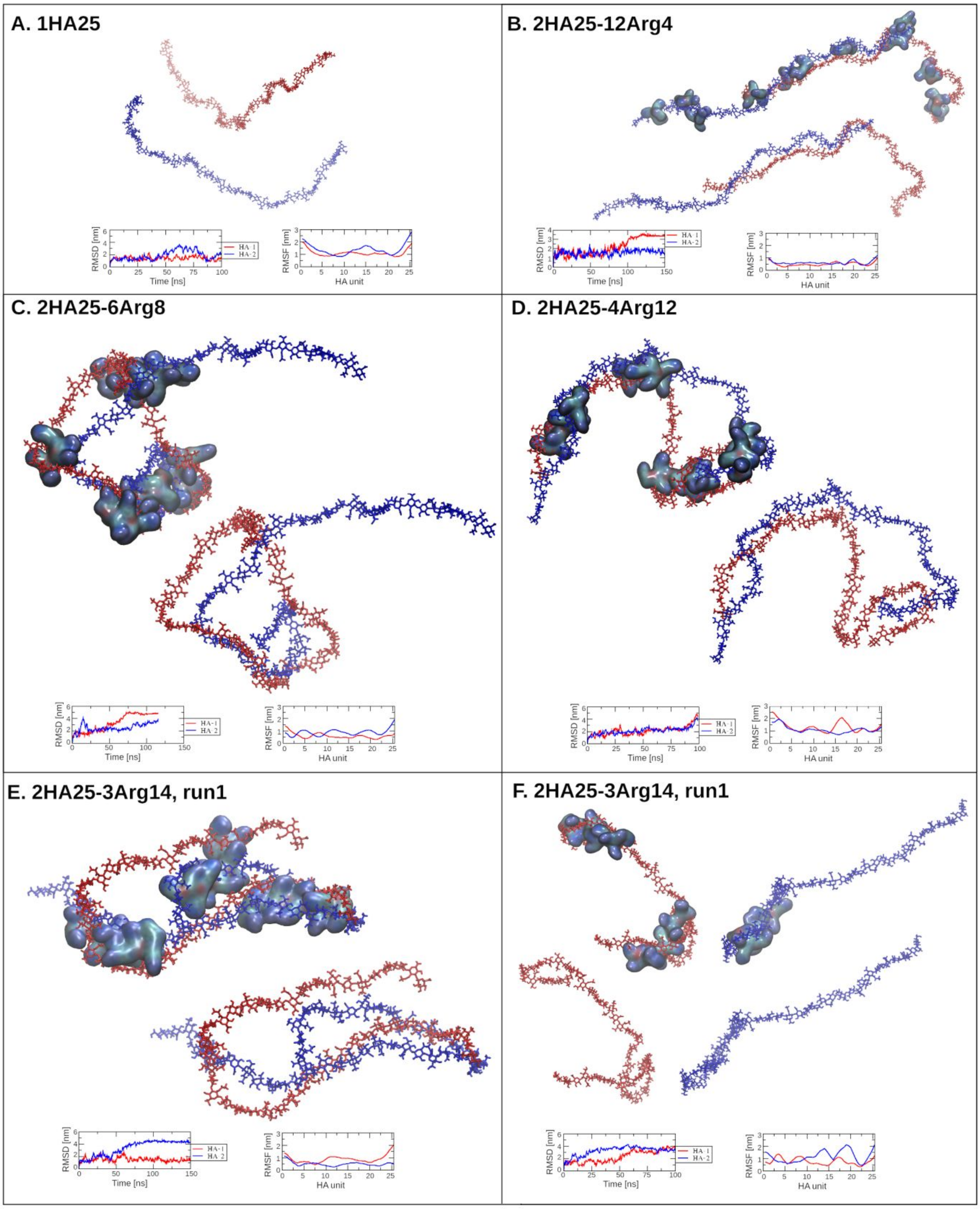

Fig. S2. Shapes of particles

A-F. Shapes are shown for the snapshots as formed at the end of the MD. One HA polymer is colored red, the other blue; polyarginine peptides are represented by molecular surface and colored according to the atom name. The upper figure in each panel represents an HA-polyarginine peptide complex (except for Fig. S2.A), the lower only HA. The RMSD and RMSF graphs of HA molecules are shown under each representation. RMSF are calculated for the stable period of MD (listed in the Table S1) with respect to the extended conformation of HA .

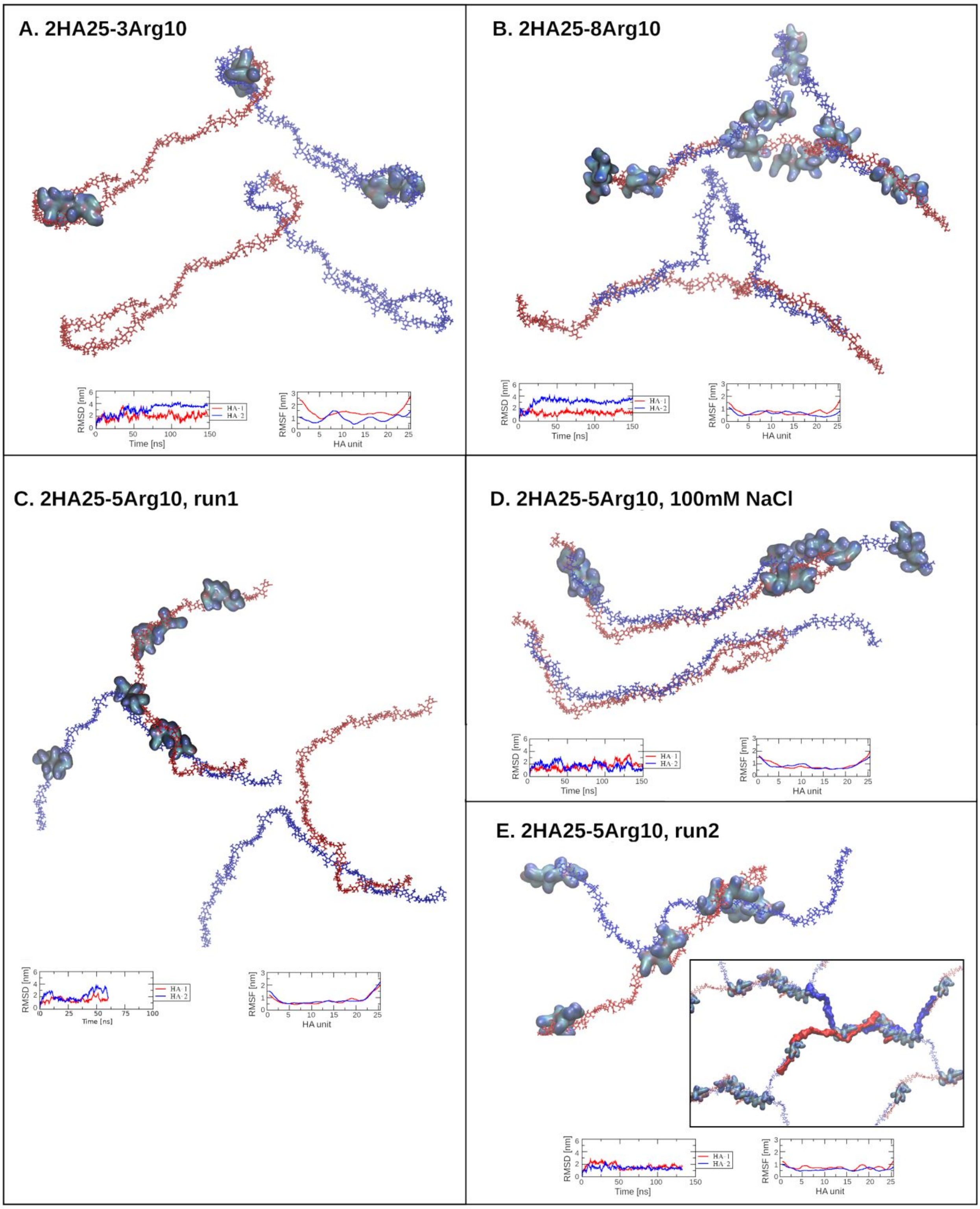

Fig. S3. Shapes of particles of the systems with Arg10

A-E. Shapes are shown for the snapshots as formed at the end of the MD. The color scheme and representation is similar to Fig. S2. Inset in Fig. S2.E shows complex networks formed in the case of the PBC problem.

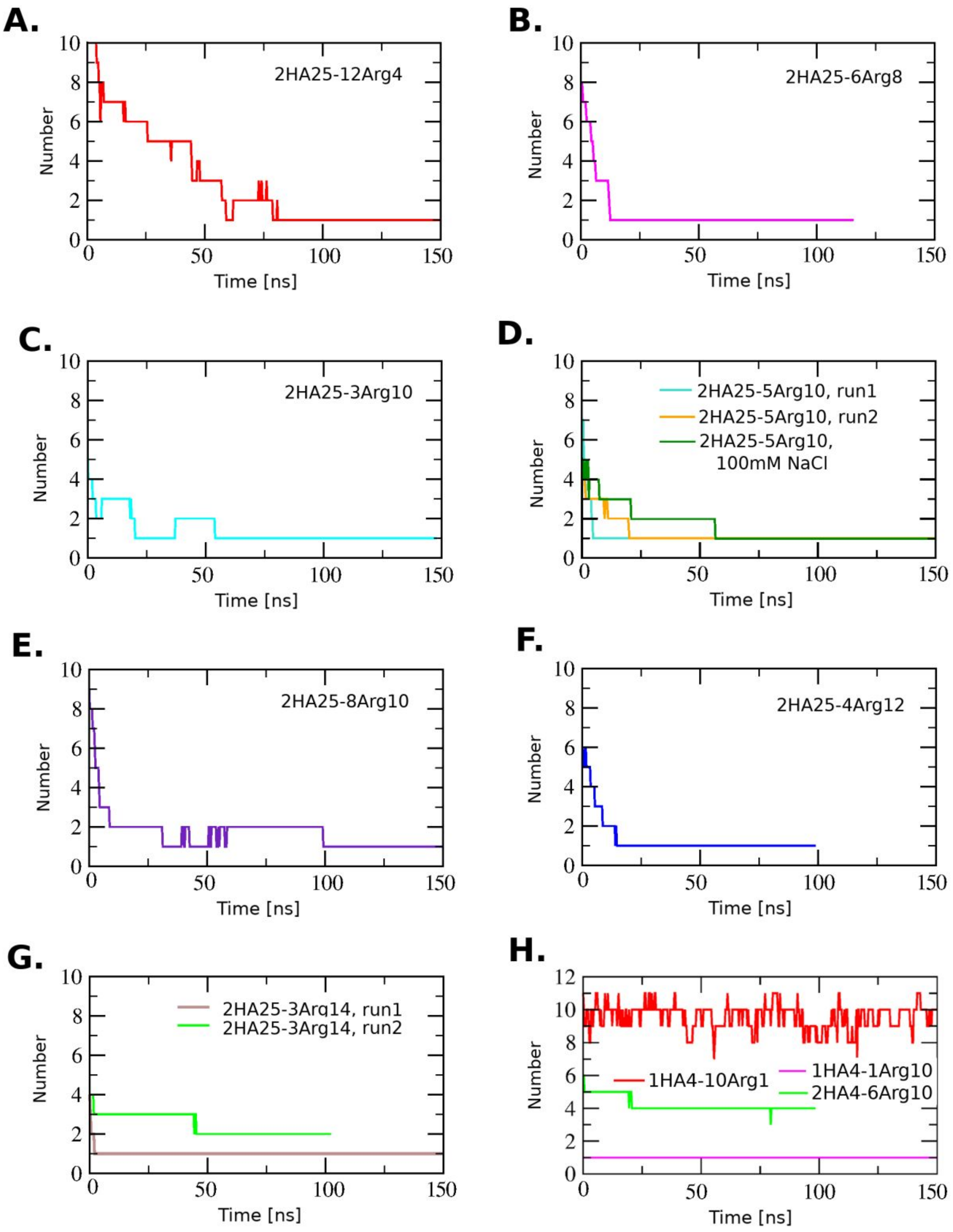

Fig. S4. Number of clusters formed during MD of polyarginine peptides-HA complexes

A. System 2HA25-12Arg4; B. system 2HA25-6Arg8; **C.** system 2HA25-3Arg10; **D.** systems 2HA25-5Arg10, simulation runs 1 (cyan) and 2 (orange) at NaCl concentration 0.01 mM, and simulation run at NaCl concentration 100mM (green); **E**. system 2HA25-8Arg10; **F.** system 2HA25-4Arg12; **G.** systems 2HA25-3Arg14, simulation runs 1 (brown) and 2 (green); **H.** systems with short HA: 1HA4-10Arg1 (red), 1HA4-1Arg10 (magenta), 2HA4-6Arg10.

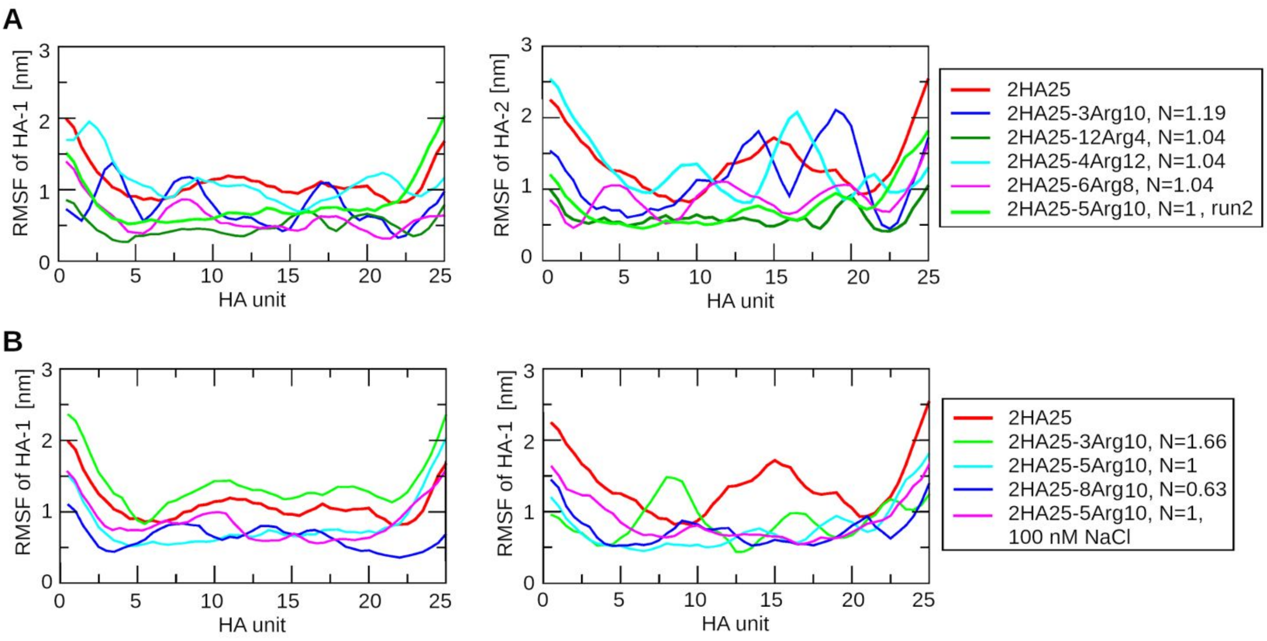

Fig. S5. RMSF of HA units of HA25 molecules

RMSF is calculated for the stable period of MD (Table S1) with respect to the structure of extended HA. There are 2 molecules of HA in each simulation, denoted as HA-1 and HA-2, each is formed by 25 HA units. The RMSF of HA molecules simulated without polyarginine peptides are also shown for comparison (red lines).

Data are split into 2 sets: **A.** Systems with different lengths of polyarginine peptide; **B.** Systems with different molar ratios[1] (N) and a similar length of polyarginine peptide (all are for MD with Arg10).

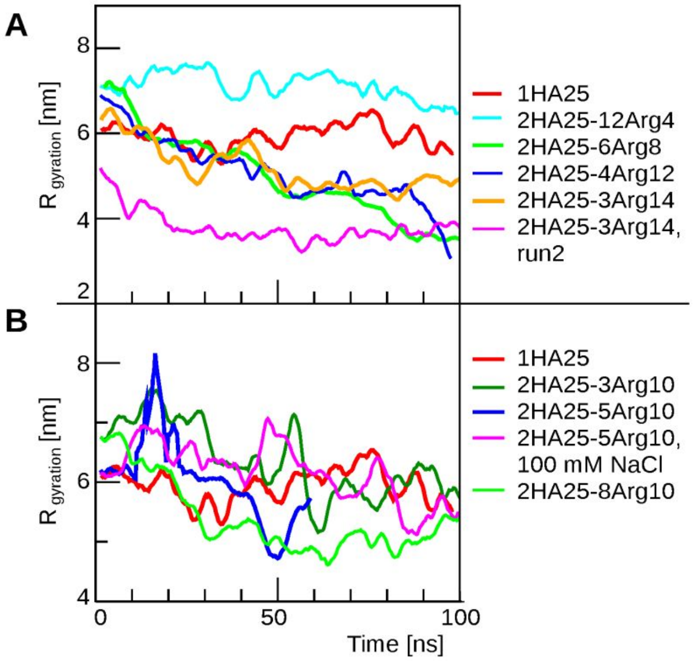

Fig. S6. The radii of gyration of HA-polyarginine peptide complexes

A. Data for representative HA with the highest radii of gyration ($R_{gyration}$) are used. Data are shown for systems with peptides of different lengths, except for Arg10. **B.** Radii of gyration ($R_{gyration}$) of HA-polyarginine peptide complexes for systems with different molar ratios, but a similar peptide length - 10Arg residues.

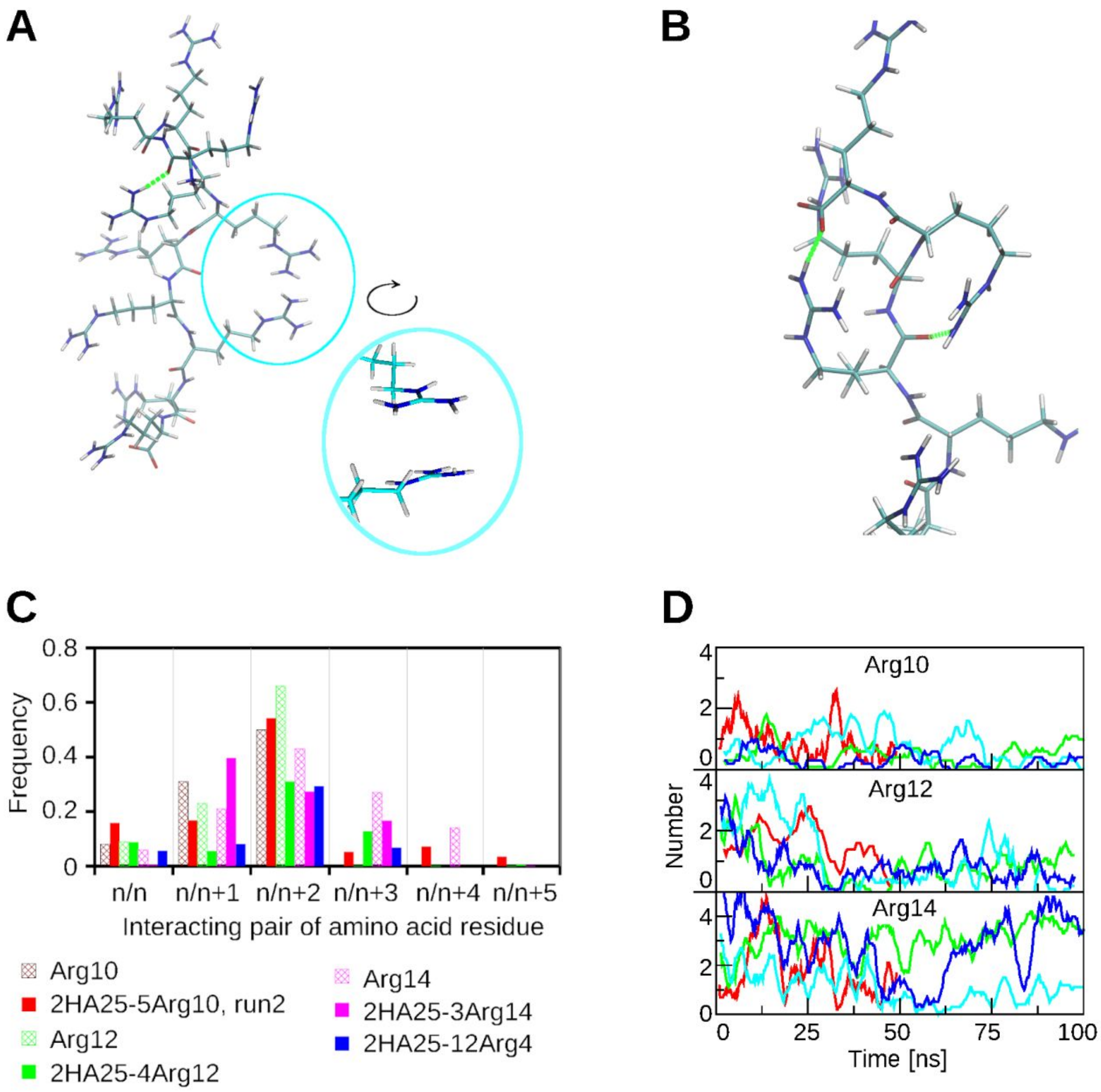

Fig. S7. Analysis of interactions between Arginines

A-B. Snapshots of polyarginines (made of 10 residues) saved from the last 5 ns of MD without HA. HB interactions between Arg residues are shown as green lines. Stacking interactions are highlighted by cyan circles and magnified in the inset. **C.** Frequency of HB formation between Arg residues with different positions in peptides, analyzed for different systems during the equilibrated period of MD. **D.** Number of internal HBs formed within the same polyarginine peptide, calculated for 10-, 12- and 14-amino-acid-long peptides during 100 ns of MD. Data from MD simulations of peptide without HA are represented by red lines; other lines (green, cyan, blue) represent 3 selected peptides from MD with 2HA25.

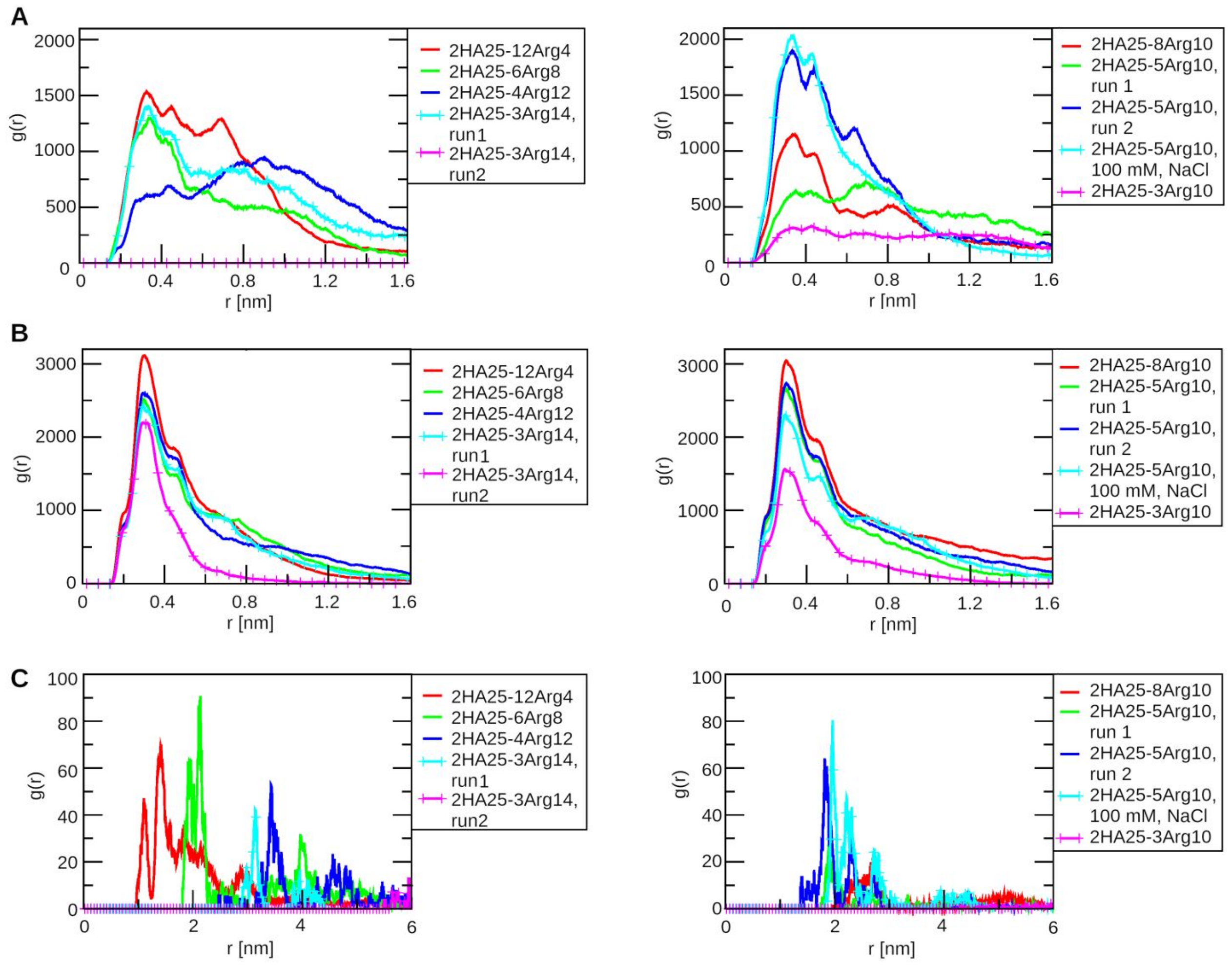

Fig. S8. RDF calculation

RDF of atoms (**A-B**) or molecules (**C**) in complexes with 2HA25. Data are averaged over 10 MD snapshots. **A.** Distribution of atoms of one HA molecule versus surface of another HA molecule; **B.** Distribution of atoms of polyarginine peptides with respect to HA surface; **C.** Distribution of center of mass of polyarginine peptides with respect to each other.

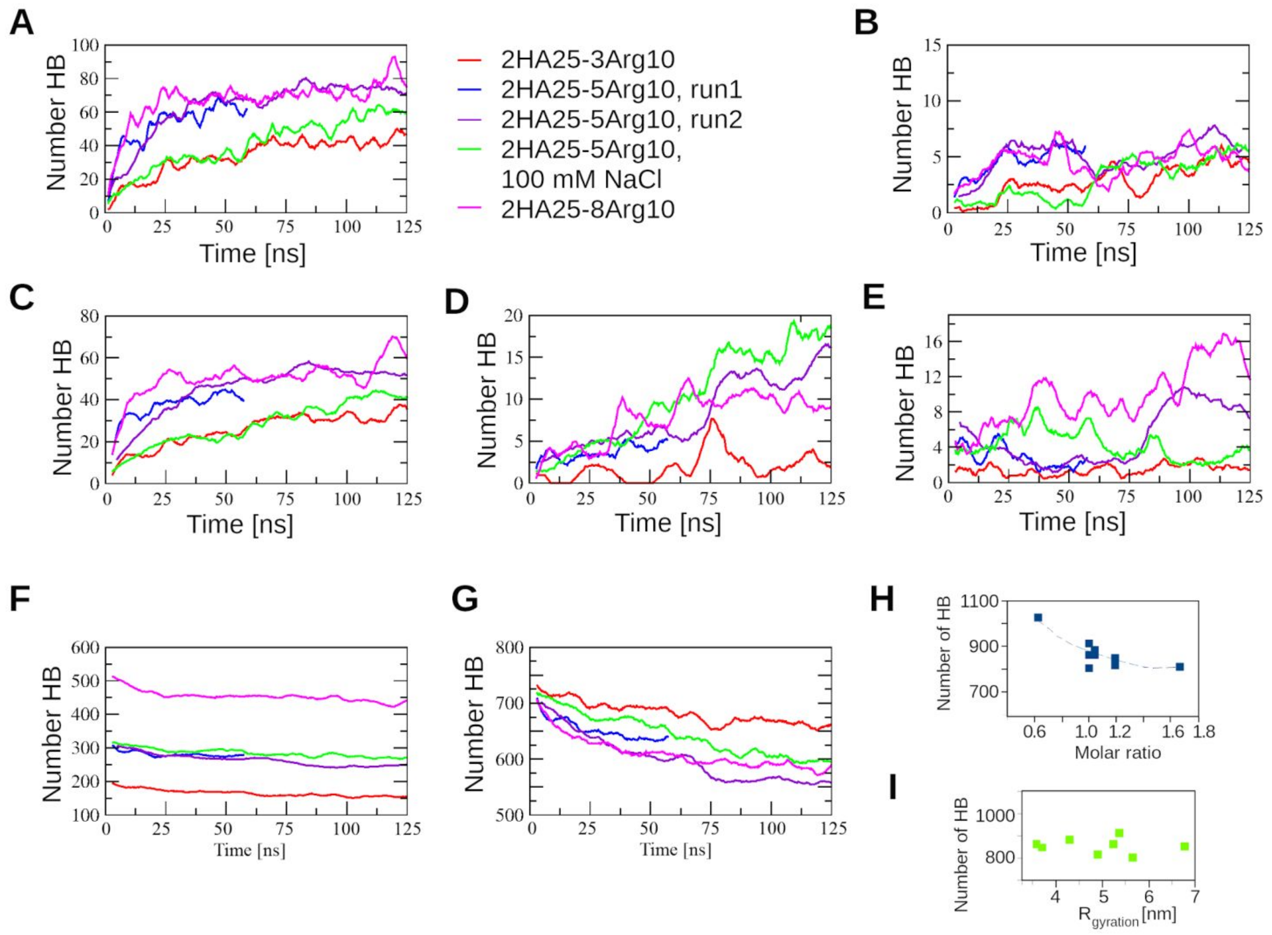

Fig. S9. Analysis of HB in HA-polyarginine systems

A-G. Number of HBs formed during MD of HA with 10-residue-long polyarginine peptides but with different molar ratios (0.625 in system 2HA25- 8Arg10, 1 in 2HA25-5Arg10, and 1.66 in 2HA25-3Arg10). Data from MD are averaged over 20 snapshots. Legend for all graphs is in **A**. **A.** Total number of hydrogen bonds between 2HA25 and polyarginine peptides. **B.** Number of hydrogen bonds between 2HA25 and main-chain of polyarginine peptides. **C.** Number of hydrogen bonds between 2HA25 and side-chain of polyarginine peptides. **D.** Number of hydrogen bonds between different HA25 polymers. **E.** Number of hydrogen bonds between polyarginine peptides (including internal bonds between residues in the same peptide). **F.** Number of hydrogen bonds between water and all polyarginine peptides. **G.** Number of hydrogen bonds between water and all HA25 polymers. **H.** Plot of the total number of HBs (formed between 2HA25-polyarginine complexes and water) versus molar ratio[1]. All systems are used for plot construction. The non-linear correlation is shown by the blue line (R^2=0.6839637). **I**. Plot of the total number of HBs (between HA-Arg complexes and water) versus $R_{gyration}$. Data are shown for 2HA25 systems with different

polyarginine peptide lengths and a molar ratio of around 1 (*i.e.* the systems 2HA25-3Arg10 and 2HA25-8Arg10 are not included).

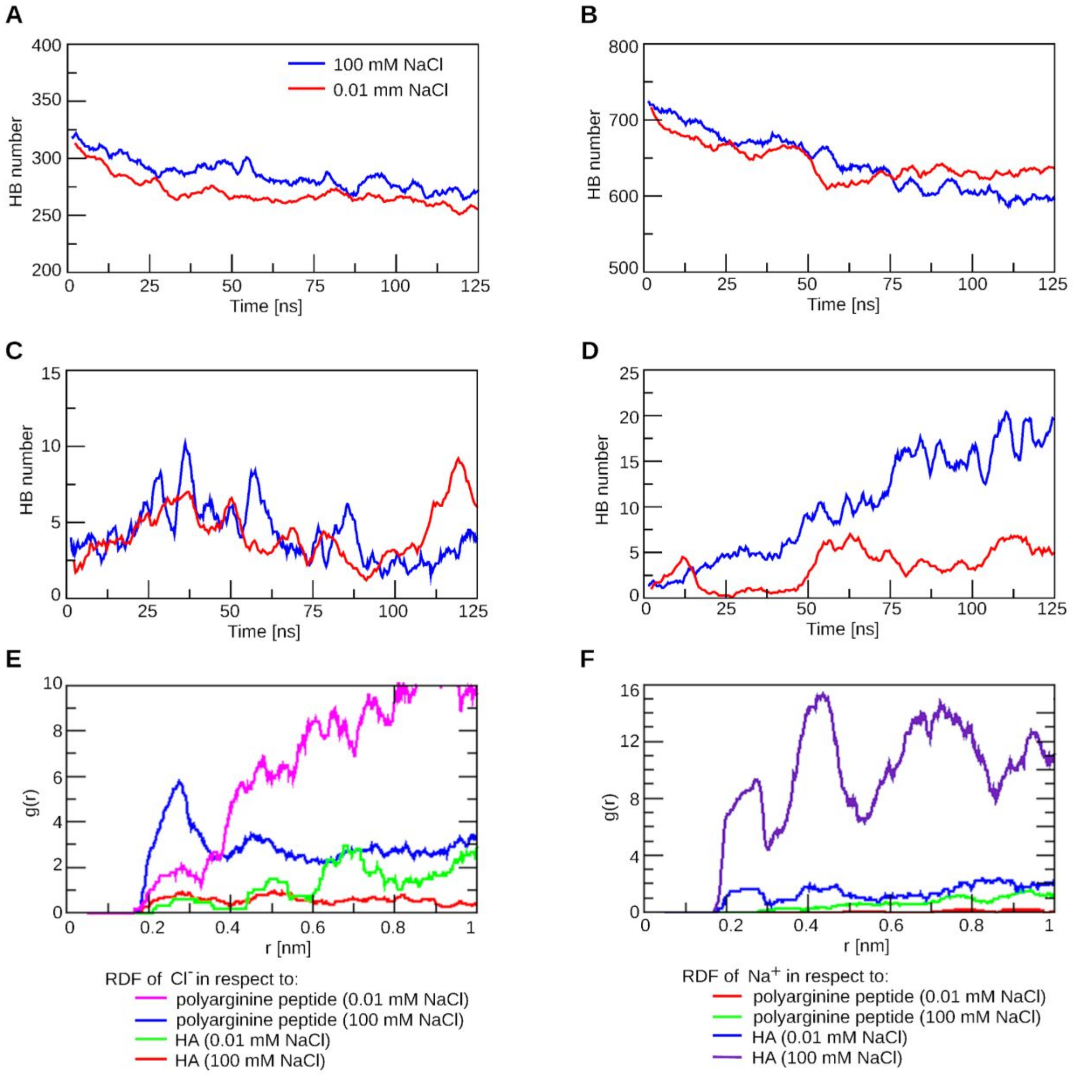

Fig. S10.Number of HBs at different concentrations of NaCl

A-D. Number of HB formed during MD of 2HA25-5Arg10 systems simulated at different concentrations of NaCl is analyzed: **A.** Between polyarginine peptides and water; **B.** Between all HA polymers and water; **C.** Between polyarginine peptides; **D.** Between HA polymers. **E.** RDF of Cl^- ions with respect to the surface of polyarginine peptide and HA. **F.** RDF of Na^+ ions with respect to the surface of polyarginine peptide and HA.

References

1. Jugl, A.; Pekař, M. Hyaluronan-Arginine Interactions-an Ultrasound and ITC Study. *Polymers - Basel.* **2020**, *12* (9). https://doi.org/10.3390/POLYM12092069.
2. Kumari, R.; Kumar, R.; Lynn, A. G-Mmpbsa -A GROMACS Tool for High-Throughput MM-PBSA Calculations. *J. Chem. Inf. Model.* **2014**, *54* (7), 1951–1962. https://doi.org/10.1021/ci500020m.
